# Some teaching resources using R with illustrative examples exploring COVID‐19 data

**DOI:** 10.1111/test.12258

**Published:** 2021-06-25

**Authors:** Arthur Berg, Nour Hawila

**Affiliations:** ^1^ Division of Biostatistics Penn State University Hershey Pennsylvania USA

**Keywords:** data science, IDSSP, maps, teaching

## Abstract

This article is presented in two parts: in the first part we discuss the use of R and R‐related tools when implementing a data science curriculum in the classroom and direct readers to helpful R resources in education, and in the second part, we demonstrate the use of R in exploring COVID‐19 data. In particular, we explore ethnic/racial distributions and COVID‐19 death rates. Supplementary R markdown files are also included allowing all graphics to be easily reproduced. It is advised that this article be discussed with sensitivity and mindfulness to potentially vulnerable students, especially as there may be students who have lost relatives due to the COVID‐19 virus.

## 1 INTRODUCTION

The International Data Science in Schools Project (IDSSP) involved a large international team of statisticians and computer scientists, together with input from several leading statistical and computer science organizations, to develop a comprehensive curriculum for introducing data science to pre‐college students or at the introductory tertiary level.

Phase 1 of the IDSSP involved developing a framework for data science curriculum, which was finished in September of 2019 and resulted in a 96‐page document published on the IDSSP website [1]. Although very detailed in terms of topic areas, methods for implementation and computing were largely left out. Phase 2, which they term the implementation phase, involves providing the basis for developing pedagogies and resources to support courses in a variety of formats that are suitable for different modes of delivery. Not much is specifically mentioned in the curriculum framework regarding specific tools that can facilitate students to apply the data science techniques discussed in the curriculum, but the following statement is provided:

Whilst no prior knowledge of software packages is assumed, students will acquire competence with at least one (freely‐available) software language or package (probably Python or R) as they progress through the course.

R and Python are both high‐level programming languages which allow users to perform a wide array of sophisticated operations with only a few, and often intuitive, lines of code, and are more suited to the classroom than, say, C. So which program—R or Python—to choose for learning data science? Here we choose R, rather than Python or any other tool. For students with just some prior learning coding experience, R may be easier to learn. Students without any prior coding experience need very, very careful scaffolding. With R, statistical models and analyses can be performed with only a few lines of code, it has a rich ecosystem of user‐contributed packages (including many teaching‐related packages), and it can generate beautiful graphics (see, e.g., the R Graph Gallery at https://www.r-graph-gallery.com). DataCamp—a resource dedicated to teaching data science online—recently posted a Reference [2] detailing many of the key differences between R and Python. However, although R might be chosen instead of Python or others for users with little to no coding experience, it is still a command‐line interface that can be daunting when trying to learn this language.

In this article, we describe why R is so much used and useful, and what R resources are currently available. We then showcase how R tools can be effectively used to explore COVID‐19 data. In particular, we explore relationships between race/ethnicity and COVID‐19 death rates in the US. Along the way, we pull together and wrangle data from different sources, make some interactive maps, and discuss various limitations/pitfalls including confounding and the ecological fallacy.

## 2 THE R PROGRAM

R is an open‐source project supported by the community developing it. It is completely free and can be easily run on computers running Windows, Mac, or Linux operating systems. It has a command line interface, and it can simply be used as a high‐functioning calculator. However, like most programming languages, R is more commonly used by writing a series of commands/functions in a source (input) file that then gets sent to the R program to be sequentially executed.

Functionality and usability of the R program is enhanced with RStudio—a freely available third‐party integrated development environment for R. RStudio also organizes a very nice library for students and teachers of R, which they call RStudio Education (https://education.rstudio.com). This site includes resources for teachers to access R related teaching materials and has details of setting up the computing infrastructure for classroom use.

At its core, R is a programming language with a command‐line interpreter. Under restrictions such as time or content, statistics teachers might shy away from teaching R syntax in their classes and instead opt for menu‐driven statistics programs such as Minitab or SPSS. However, there are now many and increasingly compelling reasons for utilizing a script for statistical analysis and data science. Most notably, scripts allow one's methods to be easily reproduced and replicated, and one does not need to navigate through a complex sequence of steps to make modifications.

Although R, as with any programming language, will be somewhat challenging to learn, early adoption of this tool will provide substantial benefits to the students in many courses outside of learning data science. In science courses like biology, physics, and chemistry, it is very common to prepare graphics and analyze data. R is a perfect tool for this, and students who have already gained familiarity with R will naturally gravitate toward this tool for their coursework and laboratory assignments. Students who gain an appreciation for data science may also seek to use R in analyzing text and various other types of discrete data they may encounter in language and other such courses.

One of the main reasons R has become so popular relates to its structure as an open source program, and this has allowed users everywhere to contribute their own features and functionality to R through packaged code referred to as R packages. Many of these packages are hosted on The Comprehensive R Archive Network (CRAN), which is directly accessible from R or RStudio. Currently, there are over 16 000 packages available on CRAN. In addition to CRAN, many users choose to host their packages on GitHub.

At the time of writing, the most trending R repository on GitHub (https://github.com/trending/r) is **swirl_courses**, which is a collection of interactive courses to use with the **swirl** R package. The swirl R package aims to teach R programming and data science interactively and directly from within the R console. There's another R package hosted on GitHub—**swirlify**—that helps instructors create content by providing tools for writing and sharing swirl courses. The swirl website (https://swirlstats.com) breaks down the steps for using swirl as a learner and as a teacher.

Tidyverse is a popular collection of powerful R packages that are widely used with statistical analyses and data science. According to the tidyverse website (https://www.tidyverse.org), “tidyverse is an opinionated collection of R packages designed for data science.” R packages included in the tidyverse collection include **tidyverse**, **ggplot2**, **dplyr**, **tidyr**, **readr**, **purrr**, and **tibble**. These packages work well with each other, hence forming the tidyverse collection. Many of these packages are used in our examples with specific usages provided in the supplementary R markdown files.

There is a fairly uniform user experience of R across the different operating systems, but there will naturally be some differences with different operating systems and different operating system versions. In a bring‐your‐own‐computer classroom setup, these subtle differences can quickly amass and pose substantial difficulties to the instructor. In addition, R is not natively supported on mobile operating systems, so students who may use a tablet as their laptop device would not be able to install R. Many of these issues are resolved using a cloud‐based service by providing a standardized user experience with access to R through a browser on any device. RStudio Cloud is an excellent cloud‐based service option for R that is particularly suited to support education. Those interested in learning more about using RStudio Cloud for education can learn more about this resource at https://education.rstudio.com/blog/2020/04/teaching-with-rstudio-cloud-q-a/.

R is the underlying programming language, and RStudio is a popular platform for interacting with R, but there is one more important R‐related concept—R markdown—that can make using R for data science very effective. R markdown is a means by which R code, R output, and annotations come together to form a dynamic, reproducible, and highly customizable document for communicating results. R markdown files can contain a narrative of the results along with displaying the explicit methods used to generate the results to allow for full transparency.

In compiling an R markdown file, R output is converted to Markdown (a lightweight markup language), which in turn is converted into an HTML, PDF, or Word document. Markdown was originally designed for HTML output, so R Markdown files that are converted to HTML output have the most flexible features compared to other formats. Some key advantages of rendering to HTML output include

- Text and other elements of the document are dynamically fitted to the browser window.
- Hyperlinks including a hyperlinked floating table of contents can allow the reader to easily move from section to section.
- Code folding allows one to collapse the technical code to minimize distraction while allowing one to see the detailed code when needed.
- Customizable CSS script can be added to the R markdown document allowing for limitless customization of the output.

If you are just getting started with R, we provide a few simple steps in the supplementary materials to allow you to easily compile your first R markdown document.

## 3 EXPLORING COVID‐19 DATA

**FIGURE 1:**
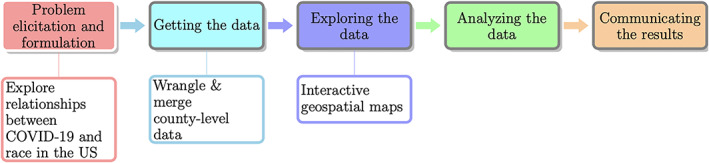
We focus on “Problem elicitation and formulation,” “Getting the data,” and “Exploring the data” steps of the basic cycle of learning from data [Colour figure can be viewed at wileyonlinelibrary.com]

We now showcase some of the many powerful features of R with illustrative examples suitable for a data science curriculum. We follow the basic cycle of learning from data [1, 3] as depicted in Figure 1, focusing just on the first three steps of “Problem elicitation and formulation,” “Getting the data,” and “Exploring the data.” We do not conduct any formal analyses or make any inferences, and we also draw attention to just some of the “worry” questions that are important in critiquing data presentations, and in teaching students to be aware of. These examples are simply meant to showcase some of data wrangling that may be required in practice, and some of the powerful features of R within the context of our motivational question.

### 3.1 Problem elicitation and formulation

The first topic of the IDSSP curriculum (Unit 1.1: *Data Science and Me*) focuses on introducing data science through discussion and case studies. The main aim of this topic is to help students become aware of the importance of data to their lives and their society. Recently, COVID‐19 has dramatically transformed the world, and with the abundance of data gathered on its spread, we can better understand how our lives are being impacted by this virus. The number of cases and deaths in various countries and regions are constantly appearing in the news. Data science can help expose the impact of COVID‐19 on society, which can be a topic of discussion with students. Here, we explore the following motivational question:

There have been media claims that there exists an association between race/ethnicity and death from COVID‐19. What data can we gather to explore these claims and what might be the problems with trying to draw conclusions?

There have been a number of reports, such as Reference [4], that highlight striking COVID‐19 racial disparities in certain parts of the US. Although these topics of race/ethnicity and COVID‐19 death rates are heavily reported in the news, it is important to discuss them with utmost sensitivity and consideration of the potential personal impacts these topics may have had on the students in the classroom, especially because race and ethnicity factors are complex and often associated with confounding variables that make drawing simple conclusions fraught. And there may be students who have lost relatives due to the virus.

One thought‐provoking question to ask students when starting this discussion is “what is the death rate for COVID‐19, and to what extent can available data help answer this question?” The answer to this question is not clear‐cut for a number of reasons:

- Medical science predicts and investigates risk factors for diseases, and for COVID‐19 have predicted increased mortality risk for patients who are older or who have underlying health conditions such as diabetes, cardiovascular disease, chronic pulmonary disease, cancer, and hypertension.
- For any given region, we do not know exactly how many people were infected by the virus. We might know the official number of confirmed cases, but there are many people who contracted the virus and were asymptomatic, couldn't get tested, or simply didn't choose to get tested.
- Especially early on in the pandemic, testing for the virus has been rather challenging. Kucirka et al[5] showed false negative rates of 38% if tests are conducted at the time symptoms just start to occur (usually 5‐6 days after contracting the virus).
- Viruses are known to mutate into different strains, and the morbidity of the different strains can vary. For example, one mutated strain of COVID‐19—the D614G mutation—has been found to be potentially more contagious though possibly less deadly, and at the time of writing, a surge of cases in Arizona was linked to this mutated form.

Deaths diagnosed to be from coronavirus are generally accurate, so the number of deaths related to COVID‐19 in a given region can generally be considered a more reliable measure than the number of cases in that region. The number of confirmed cases is often undercounted, so the proportion of coronavirus deaths to the number of cases would be an upper bound for the overall death rate of the virus. One could also compare the death rate of COVID‐19 to other contagious viruses such as the flu, SARS, MERS, H1N1, and Ebola. The viruses SARS, MERS, and especially Ebola are more deadly but easier to contain [6]. COVID‐19 has been especially detrimental due to it being easily transmitted and having little to no symptoms for some people but being quite deadly for others.

We can see that a simple question such as the death rate of the COVID‐19 cannot be easily answered, and hence it will be even more difficult to draw any conclusions about possible links between race/ethnicity and COVID‐19 death rate. Here we explore available data to get a better understanding of this topic in the aggregated ways such data are often reported. Our next step is to seek out relevant data and manipulate/transform the data so they are suitable for exploration in R.

### 3.2 Getting the data

#### 3.2.1 Datasets

There are many different possible datasets we could use and also many different analytical approaches that could be taken. Different datasets and different analyses can naturally lead to different conclusions, though datasets that are similar and analyses that are similar will often lead to similar conclusions. It is important to also note that measurement errors and other data quality issues in certain datasets, such as some urgently collected COVID‐19 datasets, can yield inferences that are not entirely accurate. The examples provided here are just meant to illustrate some fundamental concepts key to statistics and data science curricula.

In the process of forming a better understanding of the differences in COVID‐19 death rates in the US, one would likely repeatedly return to this data gathering step to continuously augment the data and consider additional avenues of exploration and analysis. We start with seeking out and combining the datasets listed below into a single dataset.

- Number of COVID‐19 deaths by county
- Racial/ethnic makeup of each county
- Map data for US counties
- Land areas of the counties
- Data corresponding states to broader US regions

It is noted that race and ethnicity data can be somewhat complex, so we use a simplified race variable and only consider the following categories: non‐Hispanic Black (subsequently referred to as Black), non‐Hispanic White (subsequently referred to as White), and Hispanic (of any race).

The New York Times started tracking COVID‐19 cases and deaths early on, and they have produced many excellent graphics with the data at https://www.nytimes.com/interactive/2020/us/coronavirus-us-cases.htm. They have also made the data publicly available through their GitHub account at https://github.com/nytimes/covid-19-data/blob/master/live/us-counties.csv, which we use for these examples. For counties that have reported over 100 deaths, the US Center for Disease Control & Prevention provides not only the number of deaths in those counties but also includes counts by race/ethnicity (https://healthdata.gov/dataset/provisional-covid-19-death-counts-county-and-race).

There are a number of ways to get race/ethnicity data by US county. The typical approach would be to access the data through the US Census Bureau website (https://www.census.gov/data/datasets/time‐series/demo/popest/2010s‐counties‐detail.html). However, these data require many manipulations/transformations to extract just the race percent‐ages by county, so instead we searched for a more straightforward dataset. This led us to the dataset *election‐context‐2018.csv*, which can be accessed from GitHub at https://github.com/MEDSL/2018-elections-unoffical. This dataset also happens to contain county‐level election data that we could integrate into the analysis should it be of interest; see for example *The Economist* article “COVID‐19 is spreading into Republican‐voting areas of America” [7].

The US Census Bureau also provides boundary data for US counties, but here again it is easier to access these data by other means. We access the data through the R package **USAboundariesData**, which includes current state, county, and congressional district boundaries as well as historical boundaries dating back to 1629. For these examples, we are just interested in the current county‐level boundaries, which we access using the function **US counties** from the **USA boundaries** package.

Land areas for each county are available from the US Census Bureau website *(* https://www.census.gov/library/publications/2011/compendia/usa-counties-2011.html#LND). Having the land areas allows us to calculate the average population density for each county; i.e. we calculate the average number of residents per square mile for each county. Finally, we also want to incorporate broad region annotations (Northeast, South, Mid‐west, and West) into the data to allow for broad comparisons of race and coronavirus impact by US region. We utilize regional mappings available on the following Kaggle website https://www.kaggle.com/omer2040/usa-states-to-region. Kaggle, a subsidiary of Google, is an online community of data scientists that allows users to find and publish data sets, explore and build models in a web‐based data science environment, work with other data scientists, and enter competitions to solve data science challenges.

Once the key datasets have been identified and procured, the next step is to wrangle the datasets into a suitable form for exploration in R.

#### 3.2.2 Data wrangling

Data wrangling is the process of manipulating/transforming raw data sources into more usable formats suitable for conducting the intended explorations and analyses. In our case, we simply wish to merge all of the datasets described above into a single “cleaned‐up” dataset with rows that correspond to different US counties and columns corresponding to different properties of each county such as the proportion of Black residents and number of COVID‐19 deaths per 10 000 residents.

Fortunately, the datasets we collected are formatted in such a way to minimize our efforts required in the data wrangling step. We first merge together the New York Times COVID‐19 dataset *us‐counties.csv* with the demographic and elections dataset *election‐context‐2018.csv* by merging on the **fips** id variable. The FIPS code gets its name from the Federal Information Processing Standard Publication 6‐4, which uniquely identifies counties and county equivalents in the US. Having a standard identification like the FIPS code is very convenient when merging datasets. We simply use the R function **merge** to merge together these datasets by the FIPS code. After merging, we find many variables are not needed. Throughout our exploration, we will make frequent use of the **select** function in the tidyverse package **dplyr** to select only the variables of interest.

The merged data includes total population and land area (in square miles) for each county. From these two variables we construct the derived variable “population density” by dividing the total population of each county by its land area. Similarly, the derived variable COVID‐19 death rate equals the number of confirmed COVID‐19 deaths per 10,000 residents, which is calculated by dividing the number of COVID‐19 deaths by the county's population then multiplying by 10 000. We will introduce other derived variables—more specifically transformed variables—later on in the examples.

### 3.3 Exploring the data

Exploring the data, seeking to describe patterns, and looking for anomalies, can help us to understand different features and relationships of variables of interest in our dataset.

#### 3.3.1 Barchart

**FIGURE 2:**
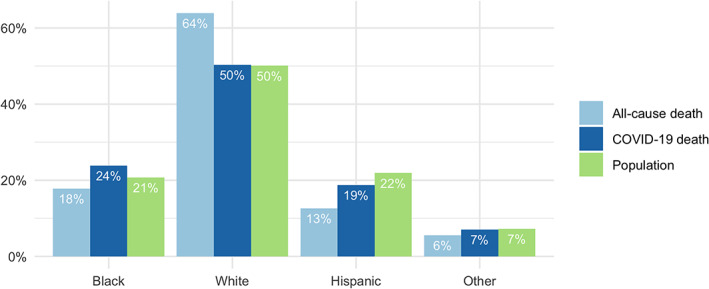
Barchart comparison of COVID‐19 deaths to all‐cause deaths by race [Colour figure can be viewed at wileyonlinelibrary.com]

We start with a comparison of COVID‐19 deaths to all‐cause deaths by race using a bar‐chart. We use a dataset from the US Center for Disease Control & Prevention, which, at the time of accessing the data (July 20, 2020), includes data from a group of 173 counties with a total of 95 538 COVID‐19 deaths of which there were 23 976 (24%) Black deaths, 50 661 (50%) White deaths, 18 901 (19%) Hispanic deaths, and 7111 (7%) other deaths. This dataset also allows us to graph the racial distribution of all‐cause deaths and the racial distribution of resident populations in this group of counties. At the time of writing, there were a total of 647 544 reported deaths (due to COVID‐19 and all other factors) in these counties since February 1, 2020 of which there are 122 302 (18%) Black deaths, 438 375 (64%) White deaths, 86 867 (13%) Hispanic deaths, and 38 274 (6%) other deaths. Figure 2 represents these percentages in an easy‐to‐interpret barchart graphic.

Putting the data in a barchart allows us to effectively visualize the comparison of COVID‐19 death rates by race/ethnicity. Here we see this dataset indicates that Black and Hispanic residents are dying of COVID‐19 at higher rates when compared to all‐cause death rates.

Summarizing the results as percent differences, we see that the COVID‐19 death rates for Black and Hispanic residents are 33% and 69% higher, respectively, than their corresponding all‐cause death rates, whereas the COVID‐19 death rate for White residents is 28% lower compared to the all‐cause death rate for White residents. It is instructive, however, to note that one should always be careful when interpreting percent difference, as it is a relative measure. For example, there is a 100% increase in going from 1% to 2%, just as there is a 100% increase in going from 50% to 100%. This is why visualizing the data in the form of a barchart, as in Figure 2, can be an effective way of summarizing data that complements simple summary measures like percent difference.

#### 3.3.2 Descriptive table

With over 3000 US counties across 50 US states, it isn't very effective summarizing the data in a table by county or by state. However, it may be informative to have summary descriptive statistics on race and coronavirus impact by the broader level of US region (Northeast, South, Midwest, and West). We use the function **aggregate** to summarize the data by region and produce Table 1.

**TABLE 1:** Comparisons by region

Variable	Northeast	South	Midwest	West
COVID‐19 deaths per 10 k residents	8.2	2.3	3.6	2.0
COVID‐19 cases per 10 k residents	115.6	111.1	82.0	89.4
% Black	11%	19%	10%	4%
% White	67%	58%	77%	51%
% Hispanic	14%	17%	7%	30%

Table 1 shows the Northeast had substantially more deaths per 10 000 residents compared to the other regions. The South and Northeast had the highest number of reported cases per 10 000 residents, and, at the time of recording these data, one would have expected these regions to have the greatest increases in deaths in the subsequent weeks.

One might have been tempted to produce Table 1 by averaging county‐level aggregated data across the four regions, but this could produce misleading statistics if the data units were counties.

Can we see anything from Table 1? It is showing that of those four broad regions, the two with the highest recorded death rates also have the greatest % of white people, and the region with the highest recorded infection rate has the second highest % both of white and of black people. It is a snapshot of records, with possibly less reliable records of cases lagging death records, only up to a particular point of time, namely, July 20, 2020. It is giving just one perspective of the impact of COVID‐19 on four broad regions of the US, up to a specific date, and more recent data may give a very different view of these broad regions.

We commented that a table of summary data for 3000 counties is not useful for trying to see patterns. The use of maps for such data is increasingly popular, so we now see the use of the geospatial visualization tools in R for the county‐level data. However, before continuing, it is valuable to help students be aware of what is sometimes called the “ecological fallacy,” which occurs when inferences about the nature of individuals are incorrectly construed from inferences about the group to which those individuals belong. A simple example of this fallacy is to make inferences about individual voter characteristics based on aggregated voter characteristics of regions. The interested reader may learn more about this in Andrew Gelman's book *Red State*, *Blue State*, *Rich State*, *Poor State* [8], in which one example is that richer US states tend to lean Democratic, but richer voters in the US tend to prefer Republican candidates. So, in exploring maps in the next section, we may be able to see and describe some more interesting patterns, but we must remember that the COVID‐19 data being considered are recorded only up to a specific date, and the data are aggregated by county.

#### 3.3.3 Maps

In moving from a very high level of aggregation to the more granular level of county aggregates, we start with visualizing the percentage of Black residents for each US county using a graded color palette with lighter colors representing higher percentages. The mapping tool we use is Leaflet, which is a widely used open‐source JavaScript library used to build web‐based maps. In particular, the package **leaflet** makes it easy to integrate and control Leaflet maps in R. Leaflet maps are actually interactive HTML maps with the ability to scroll and zoom to better visualize different parts of the US.

**FIGURE 3:**
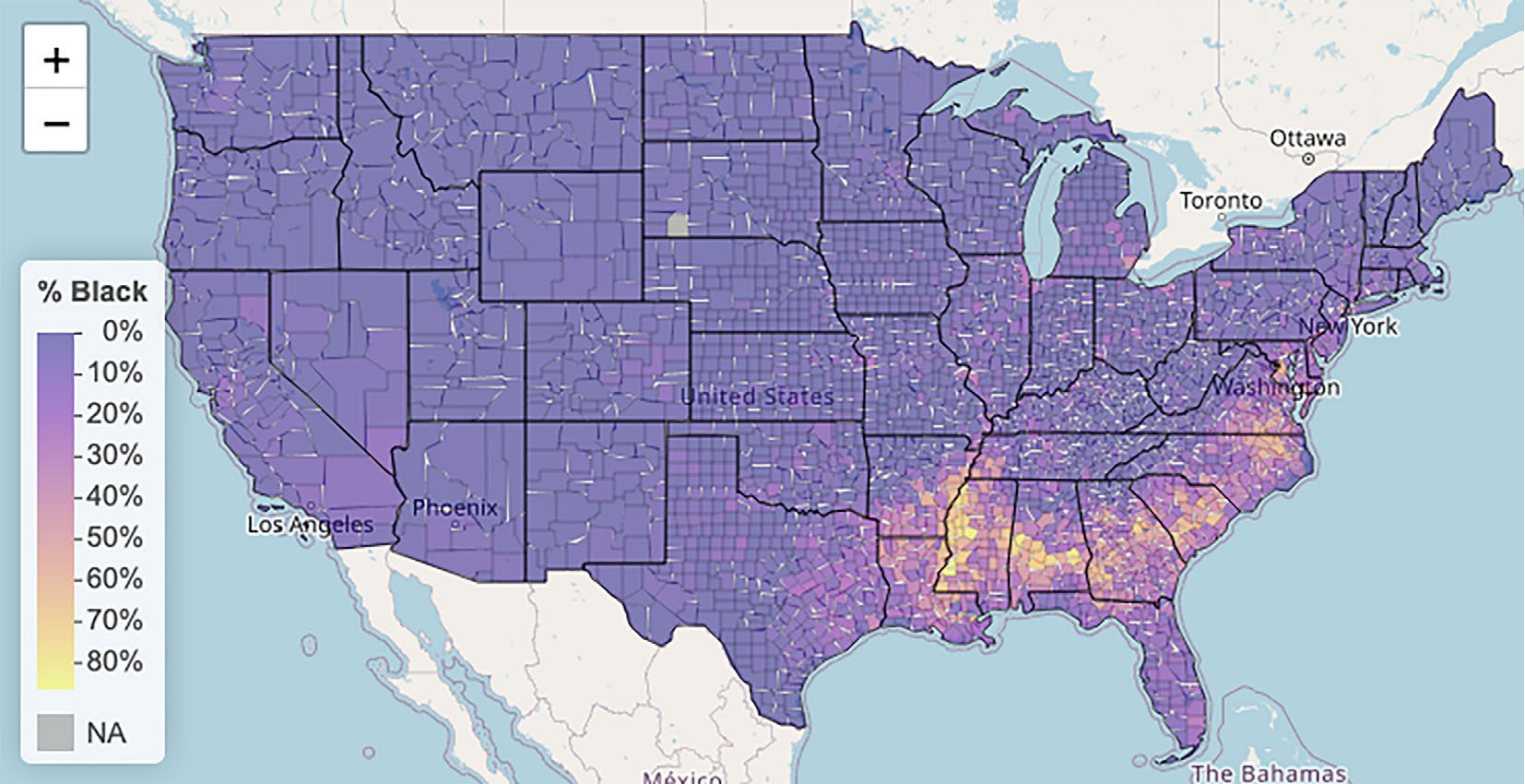
Percentage of Black residents by county [Colour figure can be viewed at wileyonlinelibrary.com]

**FIGURE 4:**
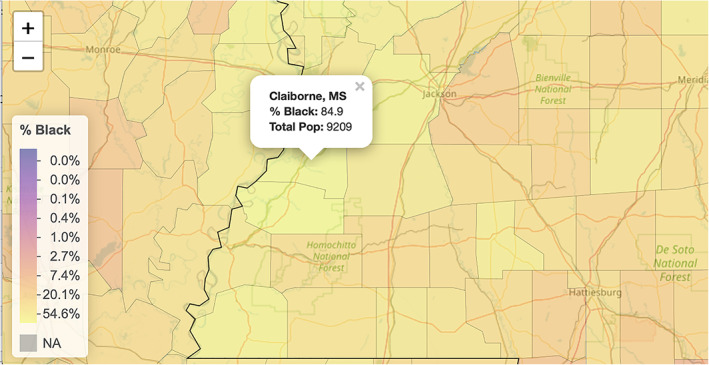
Zoomed‐in view with interactive popup [Colour figure can be viewed at wileyonlinelibrary.com]

Our first Leaflet map is Figure 3, which shows the percentage of Black residents by county. In Figure 4 we zoomed in on a few counties in Mississippi and showed the interactivity of the map by having it display various information for the county that was selected.

**FIGURE 5:**
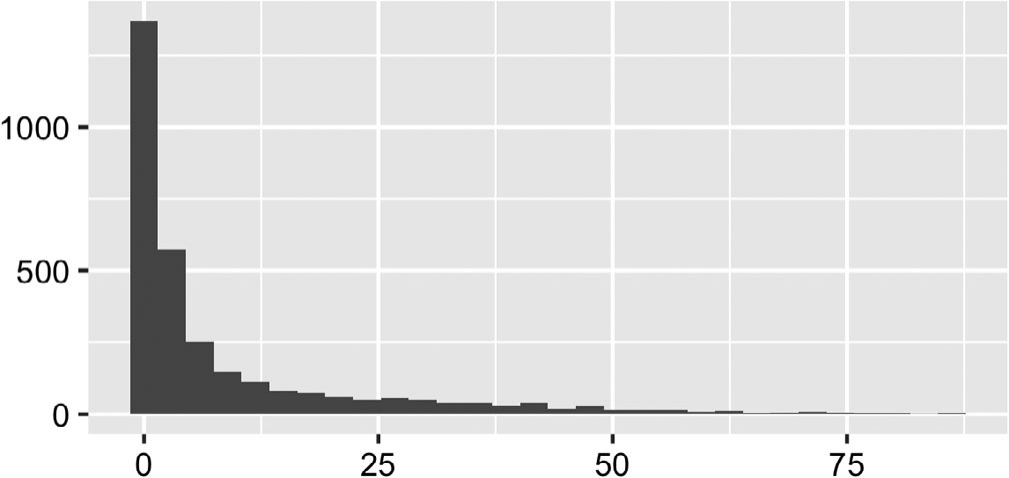
Histogram of **black_pct**

**FIGURE 6:**
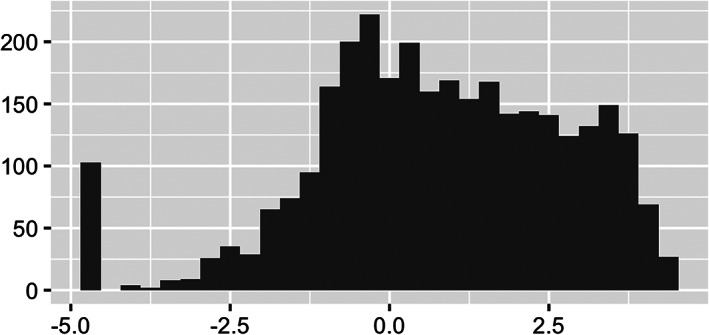
Histogram of log(**black_pct** + .01)

Especially noted in Figure 4 is the powerful use of opacity with the map coloring. The major roads and rivers that are on the map are still visible through the colors as we have set the opacity parameter to about 0.6; a value of 0 would make the colors invisible (completely transparent) and a value of 1 would make them completely non‐transparent. Careful use of opacity or alpha level as it is sometimes called can provide dramatic improvements to maps and other graphics.We see from Figure 3 that several counties in the South have high percentages of Black residents, but we are unable to differentiate Black percentages in other parts of the US. The variable **black_pct** encodes the percentages of Black residents in the 3141 US counties—this variable represents one column of our finalized dataset. Figure 5 displays a histogram of **black_pct**, and we see that this variable is highly skewed. (More specifically, you can describe it as right‐skewed or positively skewed). There are relatively few counties with very high proportions of Black residents; most of the counties have only low proportions of Black residents. We transform the **black_pct** variable with a log transformation to remove the skewness while still preserving relative orders: if *x* and *y* represent the proportions of Black residents in two counties with 0 *< x < y*, then we also have log(*x*) *<* log(*y*). As **black_pct** equals zero for several counties, and log of zero is undefined, we add the small quantity 0.01 to all of the percentages before taking the logarithms. It should be explained to students that this is an established procedure in statistical visualization (and also associated analyses) as it allows the log transformation to be used without distortion of data patterns because the quantity is so small. A histogram of the log‐transformed **black_pct** is displayed in Figure 6. In particular, we see how this transformed variable is no longer heavily skewed.

**FIGURE 7:**
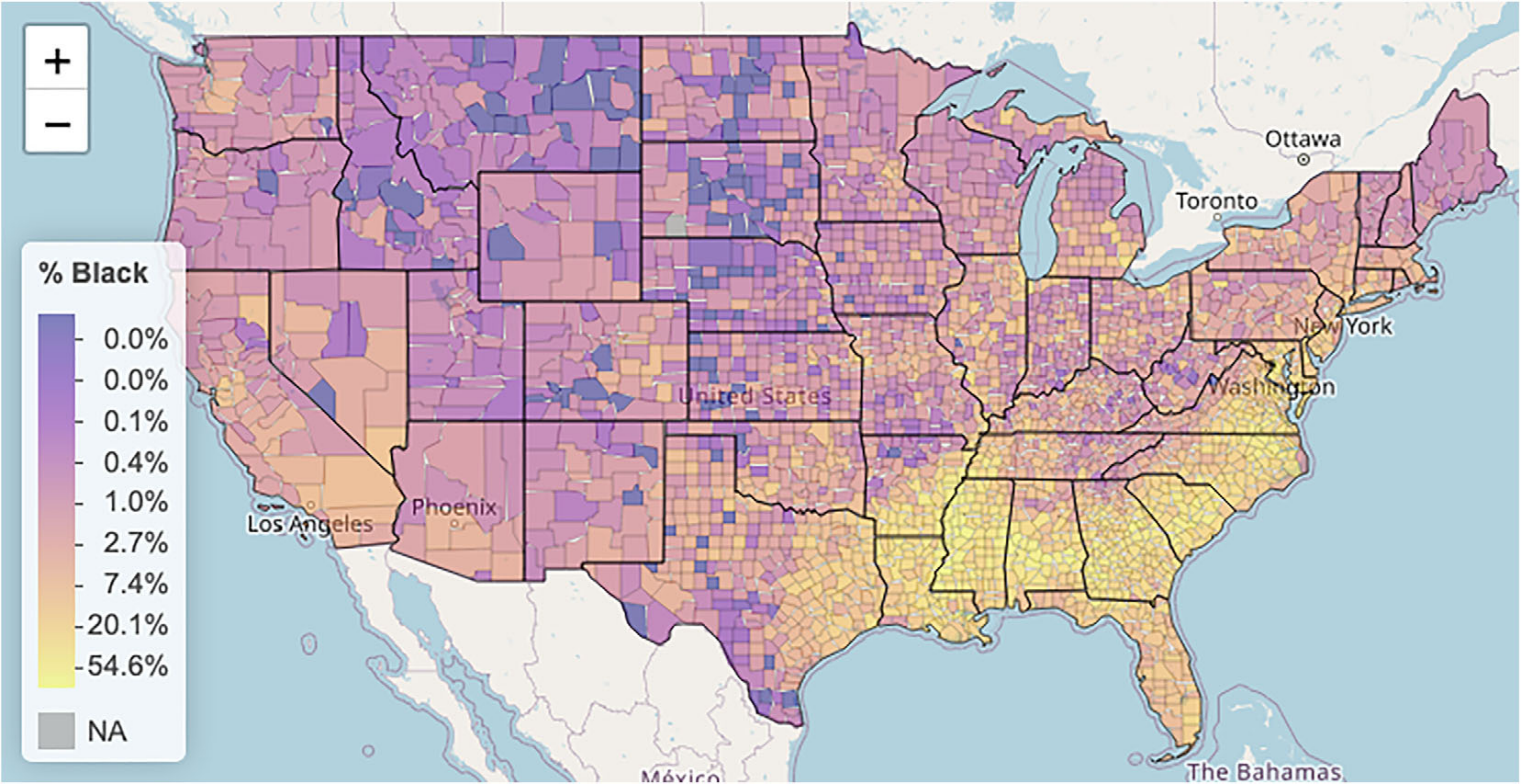
Percentage of Black residents by county with a nonlinear color scale [Colour figure can be viewed at wileyonlinelibrary.com]

Figure 7 uses the transformed **black_pct** variable when displaying the percentages of Black residents on the US map. This leads to a nonlinear scale, as depicted in the legend, but using this transformed variable allows subtle differences within each state to be more prominently displayed.

**FIGURE 8:**
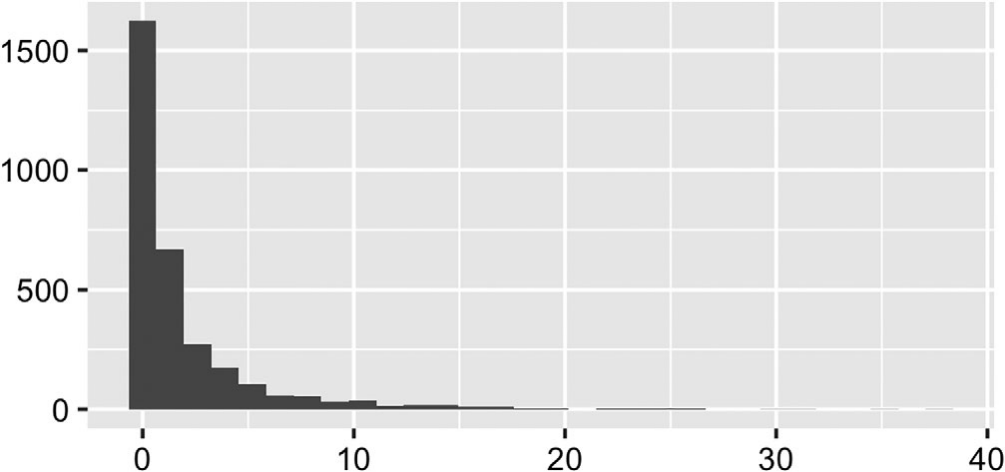
Histograms of **death rate** (deaths per 10 000 residents)

**FIGURE 9:**
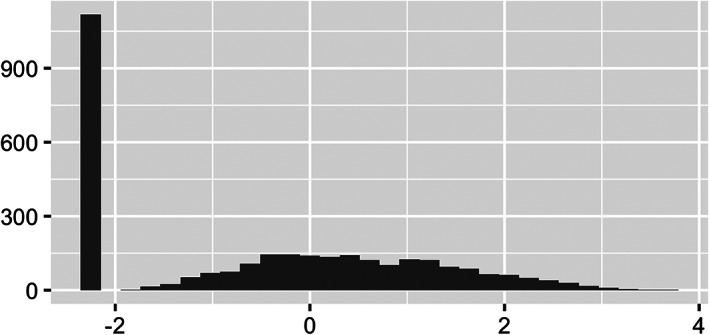
Histograms of log‐transformed **death rate**

**FIGURE 10:**
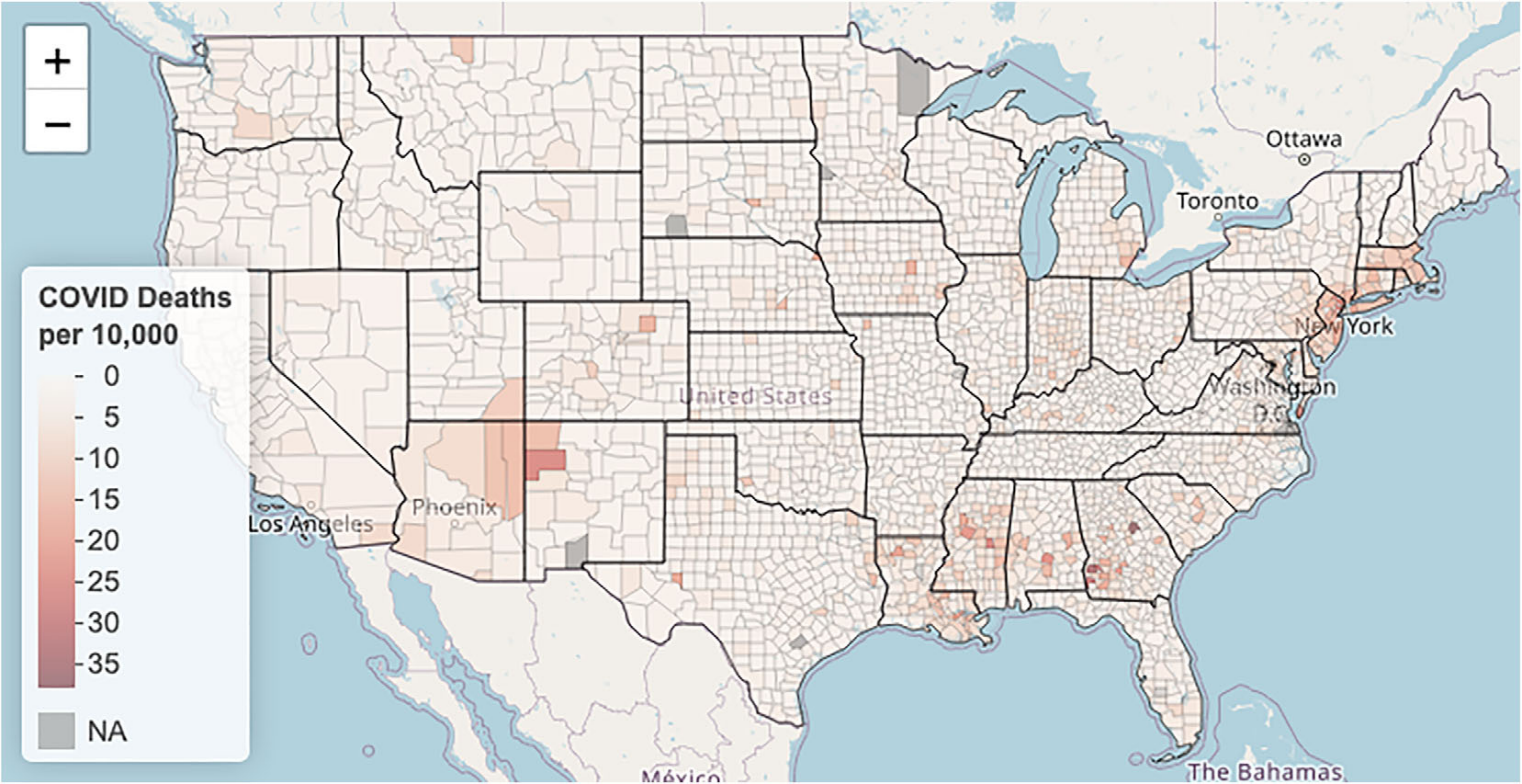
Map using **death rate** [Colour figure can be viewed at wileyonlinelibrary.com]

**FIGURE 11:**
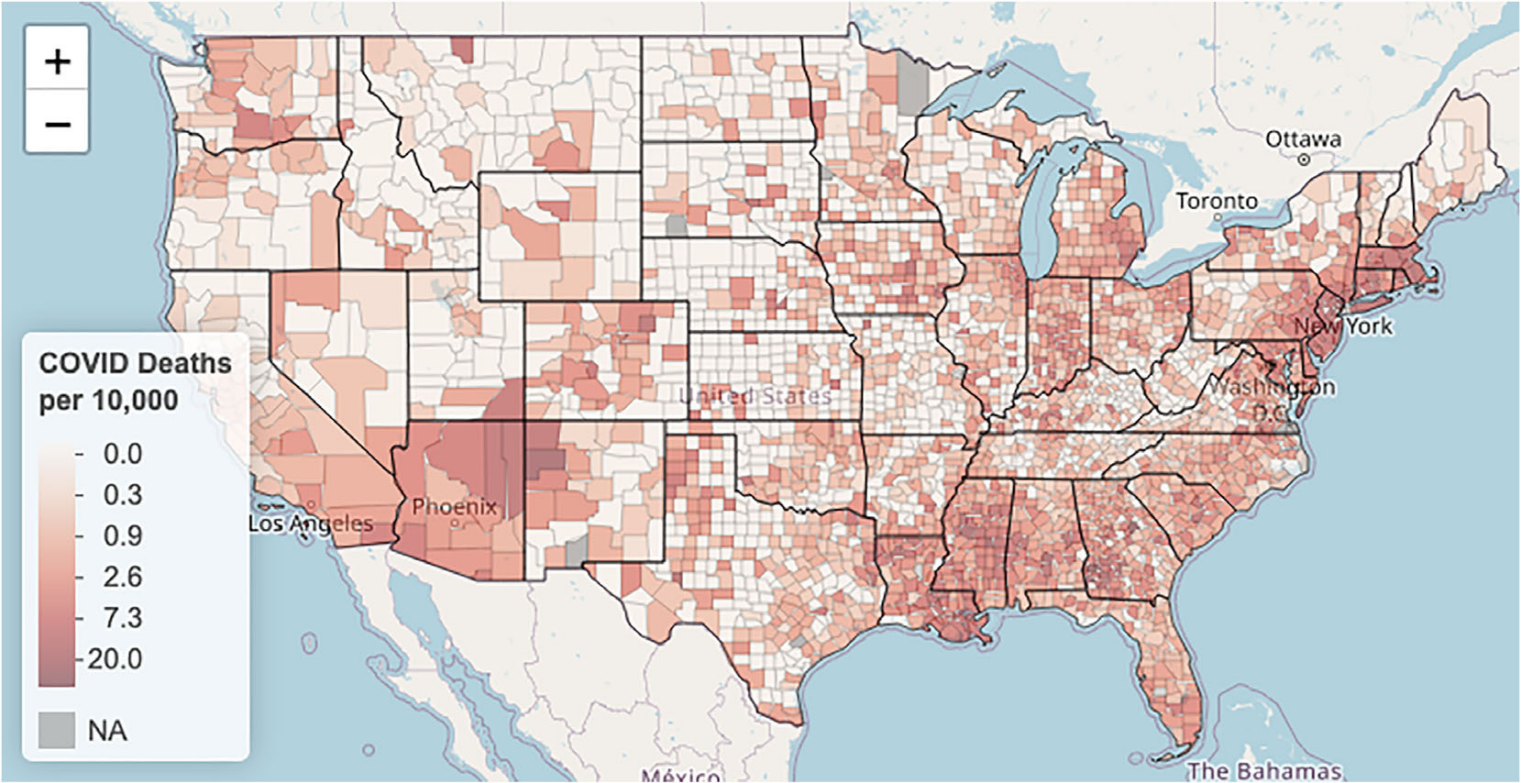
Map using log(**death rate**+0.1) [Colour figure can be viewed at wileyonlinelibrary.com]

Next, we explore COVID‐19 deaths by county; the variable **death_rate** encodes these county‐specific death rates. As previously mentioned, we consider the number of deaths per 10,000 residents so as to equally compare across different counties. Just as we observed with **black_pct**, Figure 8 shows **death rate** is also highly skewed, and we again apply a log transform to correct this skewness. Figure 9 displays a histogram of the transformed death rates and shows the resolved skewness, but it also makes it clear that many counties have no reported COVID‐19 deaths. Maps of **death rate** and log‐transformed **death rate** are presented in Figures 10 and 11, respectively. By transforming **death rate**, we have a better understanding of the relative impact of COVID‐19 on the various counties. In particular, counties in the Midwest for the most part had much lower death rates when compared to other regions of the US. The Midwest counties may be more rural, although county sizes are not necessarily determined in the same way across the US. In addition, we must remember these data are recorded up to a certain date, so COVID‐19 data patterns may need geospatial‐temporal representations.

**FIGURE 12:**
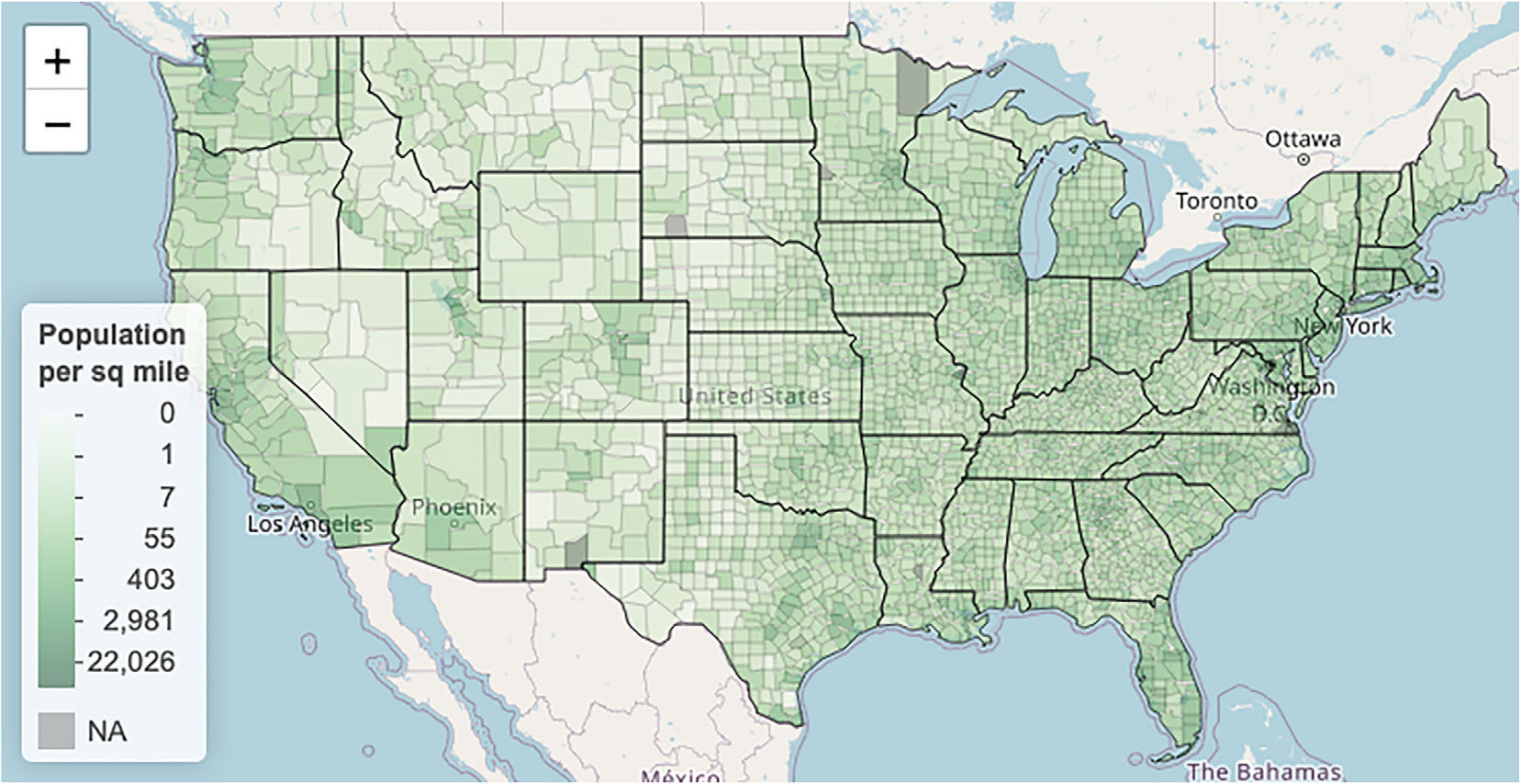
Population density by county with a non‐linear color scale [Colour figure can be viewed at wileyonlinelibrary.com]

Comparing Figures 7 and 11, we see that the spatial distribution of counties corresponding to higher percentages of Black residents is quite similar to the spatial distribution of counties corresponding to higher COVID‐19 deaths. From this, we might be tempted to jump to the conclusion that COVID‐19 is disproportionately affecting Black residents. However, there is a clear and important confounding variable that should be considered here. A confounding variable, in this case, is a variable that is related to both race and COVID‐19 deaths. Specifically, the confounding variable we are alluding to is population density. Counties that contain large cities have been observed to generally have higher COVID‐19 death rates possibly because the virus is more easily transmitted in crowded areas. Such areas may also have higher proportions of Black residents. This may also lead to considerations of income, but here we consider only population density.The variable **density** encodes the average population density of each county by dividing the county's population by its land size. Like with the other variables, **density** is also heavily skewed, so we again log‐transform this variable. Figure 12 maps the spatial distribution of population densities. Indeed, we see similar patterns as before—counties in the Midwest have a lighter color compared to counties in other parts of the US.

**FIGURE 13:**
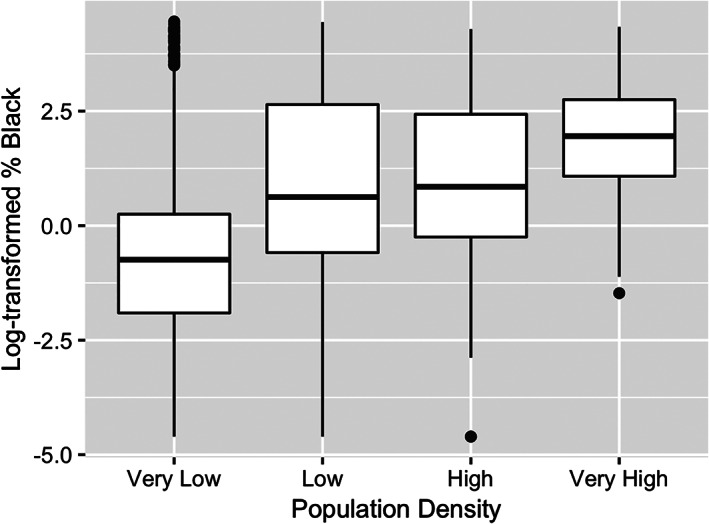
Comparison of % Black by population density levels

**FIGURE 14:**
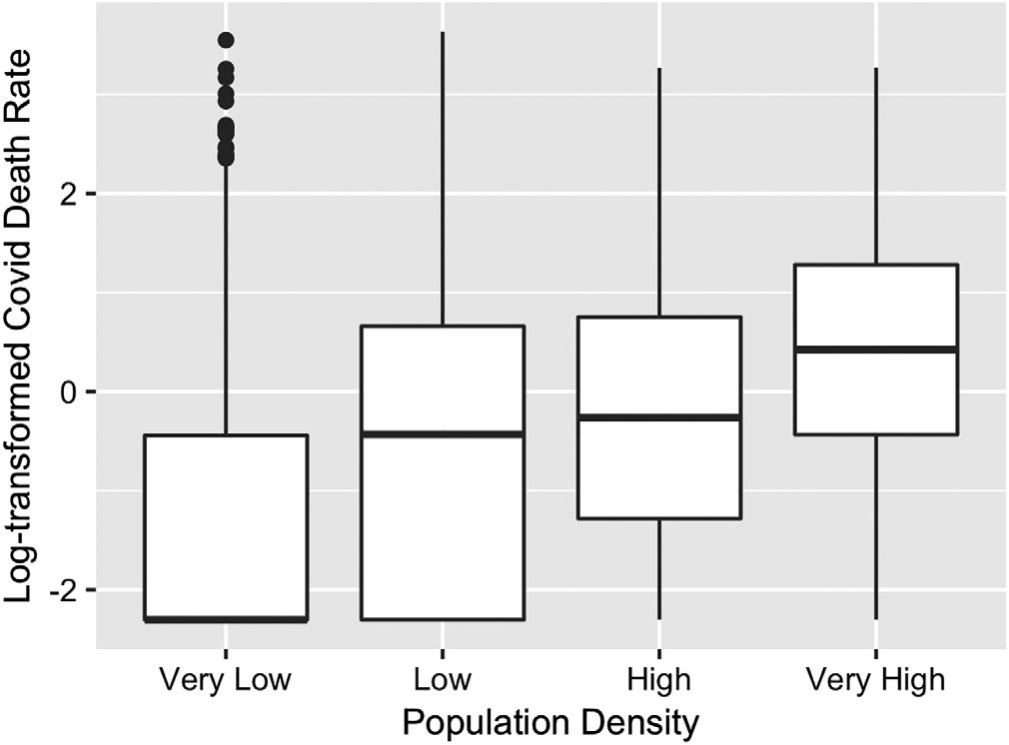
Comparison of COVID‐19 death rates by population density levels

To provide another way of seeing how population density is a confounding variable that may be related to both race and COVID‐19 death rates, we divide the counties into four groups by their population density. The counties with the lowest 25th percentile of population density are classified “Very Low,” and the counties with 25th highest percentile of population density are classified “Very High.” The classifications “Low” and “High” refer to counties in the 25th to 50th percentiles and 50th to 75th percentiles, respectively. Figures 13 and 14 use boxplots to show how both % Black and COVID‐19 death rates increase with the population density, particularly when comparing the “Very Low” to “Very High” population density levels.

## 4 FINAL REMARKS

We hope this chapter is helpful to teachers seeking to implement a data science curriculum with R. Other “easier” point‐and‐click tools may have their place in the classroom, but a truly comprehensive curriculum that best prepares students to learn data science needs to incorporate software such as R. Fortunately, as we discussed in this chapter, there are many effective resources for learning R and delivering engaging content to students.

We have used R to explore real‐world illustrative examples on a topic that has affected all of our lives—the COVID‐19 pandemic—and used statistics and data science to explore just some aspects of how its effect is seemingly different for different races/ethnicities in the US. Along the way we showed how the log transformation can improve skewness resulting in a potentially more informative graphic, we discussed ecological fallacy and confounding variables and how they challenge us to refrain from drawing any conclusions about perceived patterns, and we used many types of tables and graphs to summarize and synthesize complex datasets.

There are other ways of obtaining and wrangling data to explore possible relationships between race/ethnicity and COVID‐19 deaths in an aggregated manner as here. Epidemiological studies of such relationships require data and analysis on individuals. However, geospatial presentations of complex large real datasets are increasingly used for the exploration of aggregated data, and such explorations can lead to identification of issues and questions to be explored with more depth. Such explorations require the sourcing and wrangling of data in the civic domain and use of statistical programmes such as R; these are the learning experiences needed by students in statistics and data science.

## 5 SUPPLEMENTAL MATERIALS

A separate supplementary materials document provides step‐by‐step instructions for getting started creating R markdown documents along with student exercises to accompany this article (Appendix S1). In addition, a fully annotated R markdown document accompanies these examples as a supplementary file with more details of the functions and packages used to generate the various output presented in this manuscript (Appendix S2). The supplementary R markdown document allows all graphics included here to be easily reproduced with a single keystroke, giving full control to the reader to explore and modify as needed. All datasets and R Markdown files for this article can easily be accessed on GitHub at the following URL: https://github.com/arthurberg/Teaching_With_R. In using R Studio Cloud, customized code and annotations can be easily disseminated to the students with the instructor able to review each student's work directly within their project. We stress that good visualization and sound analyses require deep thought and hard work, so teachers should provide exercises to the students that go beyond simple cut‐and‐paste actions. Along with the illustrative examples, we also provide possible further explorations teachers can use as thought‐provoking homework exercises for the students in the supplementary materials document.

It is noted that R and the accompanying packages are constantly evolving, so it is inevitable that parts of the code will eventually become outdated and require modification. In addition, certain code and R packages may fail to work properly on different computers; this is especially true for older and outdated computers. This is the nature of working with cutting edge, constantly evolving programs.

## Supporting information

**Appendix S1.** Supporting information.

**Appendix S2.** Supporting information.
