# Supplementary material for: Some teaching resources using R with illustrative examples exploring COVID‐19 data
Source: Teach Stat. 2021 Jun 25;43(Suppl 1):S98–S109. doi: 10.1111/test.12258 (PMC8447407; doi:10.1111/test.12258)
Supplement: Supplementary file 1 — Appendix S1. Supporting information. [file TEST-43-S98-s002.pdf]

Supplementary Materials: Resources for Teachers to Use R with Illustrative Examples Exploring COVID-19 Data

1 R Markdown – Getting Started

If you are just getting started with R, you can follow these simple steps to compile your first R markdown document.

1. Install R (`https://cloud.r-project.org`).
2. Install RStudio Desktop (`https://rstudio.com/products/rstudio/download/#download`).
3. In RStudio, create a new R Markdown document: `File`▸`New File`▸`R Markdown...` .
4. You will have the choice of `Document`, `Presentation`, `Shiny` (interactive document), and `From Template` (various other examples). You can start with `Document`▸`HTML Output`. (The title and author sections can be left blank.)
5. A template R markdown file is opened. It should be saved to a directory of your choosing before it is compiled. The compiling process is called "knitting" which can be invoked from the menu or more commonly with a keyboard shortcut (Ctrl+⇧+K in Windows or ⌘+⇧+K on a Mac).
6. Now that you have compiled your first R markdown document, you're ready to start your R learning journey using the educational resources at RStudio or get started with the R Programming swirl course (`https://github.com/swirldev/swirl_courses`).

2 Student Exercises

Here we provide possible student exercises to accompany the article. Students may first try reproducing the table and graphics in the article by running the `supplement-COVID-maps.Rmd file`.

Descriptive table exercise: After loading the `df4.RData` data file in RStudio, one will be able to view the data frame `df` using the command `View`. This dataset contains many variables in addition to the ones presented in Table 1 such as presidential candidate vote totals in the 2016 presidential election, percentage of foreign born residents, percentage of residents 29 and under, percentage of residents 65 and older, and percentage of residents with an education of less than a bachelor's degree. Prepare a table by US region, similar to Table 1, but with descriptive statistics presented on these other variables. It is important to note that, before aggregating the data by region, the county-level percentages should be converted to counts by multiplying by the respective county's total population. This will avoid issues such as the "ecological fallacy" as described in the article.

Political party exercise: In this exercise we explore the presidential candidate vote totals in the 2016 presidential election. The front runner candidates in that election were Donald Trump (Republican) and Hillary Clinton (Democrat). The number of votes for these candidates are encoded in the variables `trump16` and `clinton16`. Define a `republican` variable to be `trump16/(trump16+clinton16)`. Draw a histogram of this variable and determine if it is heavily skewed liked the other variables considered in this section. See if a log transform makes the histogram more or less skewed. Map the `republican` proportion data on a US map and make it interactive so that selecting

any particular county will produce the republican proportion along with the number of Trump and Clinton votes. Finally explore the relationship between `republican` and log-transformed COVID-19 death rate and explore the potential confounding relationship with population density.
