# Supplementary material for: Some teaching resources using R with illustrative examples exploring COVID‐19 data
Source: Teach Stat. 2021 Jun 25;43(Suppl 1):S98–S109. doi: 10.1111/test.12258 (PMC8447407; doi:10.1111/test.12258)
Supplement: Supplementary file 2 — Appendix S2. Supporting information. [file TEST-43-S98-s001.pdf]

Illustrative Examples: COVID-19 and Race/Ethnicity in the US – Maps

February 15, 2021

Contents

Getting things setup **1**
Install/Load Packages 1
Setting the Working Directory 2
The Datasets 2
Data Wrangling 2

Barchart (Figure 1) **3**

Proportions of Non-Hispanic Blacks **6**
Histograms (Figure 4) 6
Maps (Figures 3 and 5) 7

Prevalence of COVID-19 Deaths **9**
Histograms (Figure 6) 9
Maps (Figure 7) 10

Population Density **12**
Adding land area variable 12
Histograms 12
Maps (Figure 8) 13
Boxplot 15

Getting things setup

Install/Load Packages

The following code installs a set of R packages used in this document – if not already installed – and then loads the packages into R. Note that we utilize the US CRAN repository, but other repositories may be more convenient according to geographic location.

```
list.of.packages <- c("dplyr", "knitr", "devtools", "ggplot2",
    "patchwork", "Hmisc", "gtsummary", "gt", "leaflet",
    "sf")
new.packages <- list.of.packages[!(list.of.packages %in%
    installed.packages()[, "Package"])]
if (length(new.packages)) install.packages(new.packages,
    repos = "http://cran.us.r-project.org")
lapply(list.of.packages, require, character.only = TRUE)
# remotes::install_github('rstudio/webshot2',quiet=T)
devtools::install_github("ropensci/USAboundaries", quiet = T)
devtools::install_github("ropensci/USAboundariesData", quiet = T)
```

```
# library(webshot2) library(mapview)
library(USAboundaries)
```

Setting the Working Directory

Make sure the working directory for RStudio is in the appropriate location with the set working directory (`setwd`) command.

```
setwd("~/Box/Teaching Statistics Special Issue/4th resubmission/software")
```

The Datasets

This analysis integrates datasets from a few different repositories including the US Centers for Disease Control and Prevention, the US Census Bureau, and GitHub. In particular we will be using the following datasets.

- The New York Times started tracking COVID-19 cases and deaths early on and they produce many excellent graphics with the data at https://www.nytimes.com/interactive/2020/us/coronavirus-us-cases.html. They also have made the data publicly available through their GitHub account at https://github.com/nytimes/covid-19-data/blob/master/live/us-counties.csv. This GitHub data is used for these examples.
- The MIT Election Data + Science Lab has links to many election-related datasets (https://electionlab.mit.edu/data). The dataset we use is `election-context-2018.csv`, accessed from GitHub at https://github.com/MEDSL/2018-elections-unoffical.
  - This dataset contains county-level results of the 2016 presidential election along with other county-level data including race, ethnicity, and various other socioeconomic variables.

Data Wrangling

Data wrangling is the process of manipulating/transforming raw data sources into more useable formats that is more suitable for conducting the intended analysis. In our case we simple wish to appropriately merge all of the datasets described above into a single "cleaned-up" dataset. Ultimately we want a single dataset with rows that correspond to different US counties with data on each county organized in the various columns. This can be a somewhat tedious part of data science, and rarely a single best approach. Many different approaches are available, and these tools will become more familiar with practice. Below we provide code for how we conducted the data wrangling, but as we do not intend to put emphasis on these steps, we simple present the code without much description.

Load and merge COVID & race data

Two of the datasets are a CSV (comma-seperated values) file, which is a fairly standard format. We will use the built-in R function `read.csv` to load in the data. An alternative "tidyverse" method to load the data is with the `read_csv` command in the `readr` package. After loading the datasets, they are merged on the common `fips` variable. The resulting merged dataset is called `df`, standing for "data frame".

```
data.covid <- read.csv("Data/us-counties.csv")
data.demographics <- read.csv("Data/election-context-2018.csv")
df = merge(data.covid, data.demographics, all = TRUE, by = "fips")
```

The merged data contain more variables than we are interested in analyzing, so we keep only the variables of interest using the `select` function in the `dplyr` package. Note here is the first of many times we use the pipe function (%>%). More about how the pipe function works is described at https://magrittr.tidyverse.org.

```
df2 = df %>% dplyr::select(fips, cases, deaths, total_population,
    black_pct, white_pct, hispanic_pct, rural_pct)
```

Add in the US county map data

US county map data is obtained through the R function `us_counties` in the `USAboundaries` package. We then merge the US county map data with the previously merged datasets into `df3`.

```
map_counties <- USAboundaries::us_counties()
map_counties$fips = as.numeric(map_counties$geoid)
df3 = merge(map_counties, df2, all.x = TRUE, by = "fips")
```

Barchart (Figure 1)

- County level data (including proportion from COVID related death and all cause related death) on race and Hispanic origin is available for counties with more than 100 COVID-19 deaths. We perform some data cleaning and create a new dataframe `cdc.diff.pct` that includes county-level data for each race on the difference between the proportion from all-cause deaths and proportion from COVID related deaths
- A table is produced that shows the total (for 173 counties with more than 100 deaths) number of all cause deaths, COVID deaths and population
- The following code is also used to reproduce Figure 1 of the manuscript which compares proportions of COVID-19 deaths to all-cause deaths by race

```
cdc <- read.csv("Data/Provisional_COVID-19_Death_Counts_by_County_and_Race.csv")
cdc <- dplyr::rename(cdc, c("fips"="FIPS.Code"))
cdc=merge(cdc,select(df3,total_population, fips), by="fips")

cdc$Indicator[cdc$Indicator=="Distribution of all-cause deaths (%)"]<-"allcause"
cdc$Indicator[cdc$Indicator=="Distribution of COVID-19 deaths (%)"]<-"covid"
cdc$Indicator[cdc$Indicator=="Distribution of population (%)"]<-"population"


cdc2=cdc[, c("COVID.19.Deaths.Total",
             "All.Deaths.Total",
             "total_population",
             "Non.Hispanic.Black",
             "Non.Hispanic.White",
             "Hispanic",
             "Non.Hispanic.American.Indian.or.Alaska.Native",
             "Non.Hispanic.Asian",
             "Other",
             "Indicator")]

cdc2.allcause=cdc2[cdc2$Indicator=="allcause",]
cdc2.covid=cdc2[cdc2$Indicator=="covid",]
cdc2.population=cdc2[cdc2$Indicator=="population",]

cdc2.allcause.count <- round(apply(cdc2.allcause[,
  c("Non.Hispanic.Black",
    "Non.Hispanic.White",
    "Hispanic",
    "Non.Hispanic.American.Indian.or.Alaska.Native",
    "Non.Hispanic.Asian",
    "Other")] *
    as.numeric(gsub(",", "", cdc2.allcause$All.Deaths.Total)),2,sum))
```

```r
cdc2.covid.count <- round(apply(cdc2.covid[,
  c("Non.Hispanic.Black",
    "Non.Hispanic.White",
    "Hispanic",
    "Non.Hispanic.American.Indian.or.Alaska.Native",
    "Non.Hispanic.Asian",
    "Other")] *
    as.numeric(gsub(",", "", cdc2.covid$COVID.19.Deaths.Total)),2,sum))

cdc2.population.count <- round(apply(cdc2.covid[,
    c("Non.Hispanic.Black",
      "Non.Hispanic.White",
      "Hispanic",
      "Non.Hispanic.American.Indian.or.Alaska.Native",
      "Non.Hispanic.Asian", "Other")] *
      as.numeric(gsub(",", "", cdc2.population$total_population)),2,sum))



cdc2.allcause.count2=cdc2.allcause.count[
  c("Non.Hispanic.Black",
    "Non.Hispanic.White",
    "Hispanic")]
cdc2.allcause.count2["Other"] = sum(cdc2.allcause.count[
  c("Non.Hispanic.American.Indian.or.Alaska.Native",
    "Non.Hispanic.Asian",
    "Other")])

cdc2.covid.count2=cdc2.covid.count[
  c("Non.Hispanic.Black",
    "Non.Hispanic.White",
    "Hispanic")]
cdc2.covid.count2["Other"] = sum( cdc2.covid.count[
  c( "Non.Hispanic.American.Indian.or.Alaska.Native",
     "Non.Hispanic.Asian",
     "Other")])

cdc2.population.count2=cdc2.population.count[
  c("Non.Hispanic.Black",
    "Non.Hispanic.White",
    "Hispanic")]

cdc2.population.count2["Other"] = sum(cdc2.population.count[
  c("Non.Hispanic.American.Indian.or.Alaska.Native",
    "Non.Hispanic.Asian",
    "Other")])

cdc2.allcause.prop=cdc2.allcause.count2/sum(cdc2.allcause.count2)
cdc2.covid.prop=cdc2.covid.count2/sum(cdc2.covid.count2)
cdc2.population.prop=cdc2.population.count2/sum(cdc2.population.count2)
```

```r
df6 <- reshape2::melt(cbind(data.frame(cdc2.allcause.prop), data.frame(cdc2.covid.prop), data.frame(cdc2
df6$race <- rep(c("Black", "White", "Hispanic","Other"), 3)

tab=cbind(data.frame(cdc2.allcause.count2), data.frame(cdc2.covid.count2), data.frame(cdc2.population.co

kable(tab)
```

	cdc2.allcause.count2	cdc2.covid.count2	cdc2.population.count2
Non.Hispanic.Black	122302	23976	30771353
Non.Hispanic.White	438375	50661	74467710
Hispanic	86867	18901	32566653
Other	38274	7111	10819018

```r
colnames(df6) <- c("Variable", "Proportion", "Race")

library(plyr)
df6$Variable=mapvalues(df6$Variable,from=
                        c("cdc2.allcause.prop",
                          "cdc2.covid.prop",
                          "cdc2.population.prop"),
                      to=c("All-cause death",
                           "COVID-19 death",
                           "Population"))
df6$Race=factor(df6$Race,levels=c("Black", "White", "Hispanic", "Other"))

gr11=
  ggplot(df6,aes(Race,Proportion,fill=Variable)) +
  geom_bar(stat="identity",position='dodge') +
  labs(x="", y="") +
  geom_text(aes(
    label=scales::percent(Proportion,accuracy=1)),
    vjust=1.6,
    color="white",
    position = position_dodge(0.9),
    size=3) +
  scale_fill_brewer(palette="Paired") +
  theme_minimal() +
  theme(legend.title = element_blank()) +
  scale_y_continuous(labels=scales::percent)

gr11
```

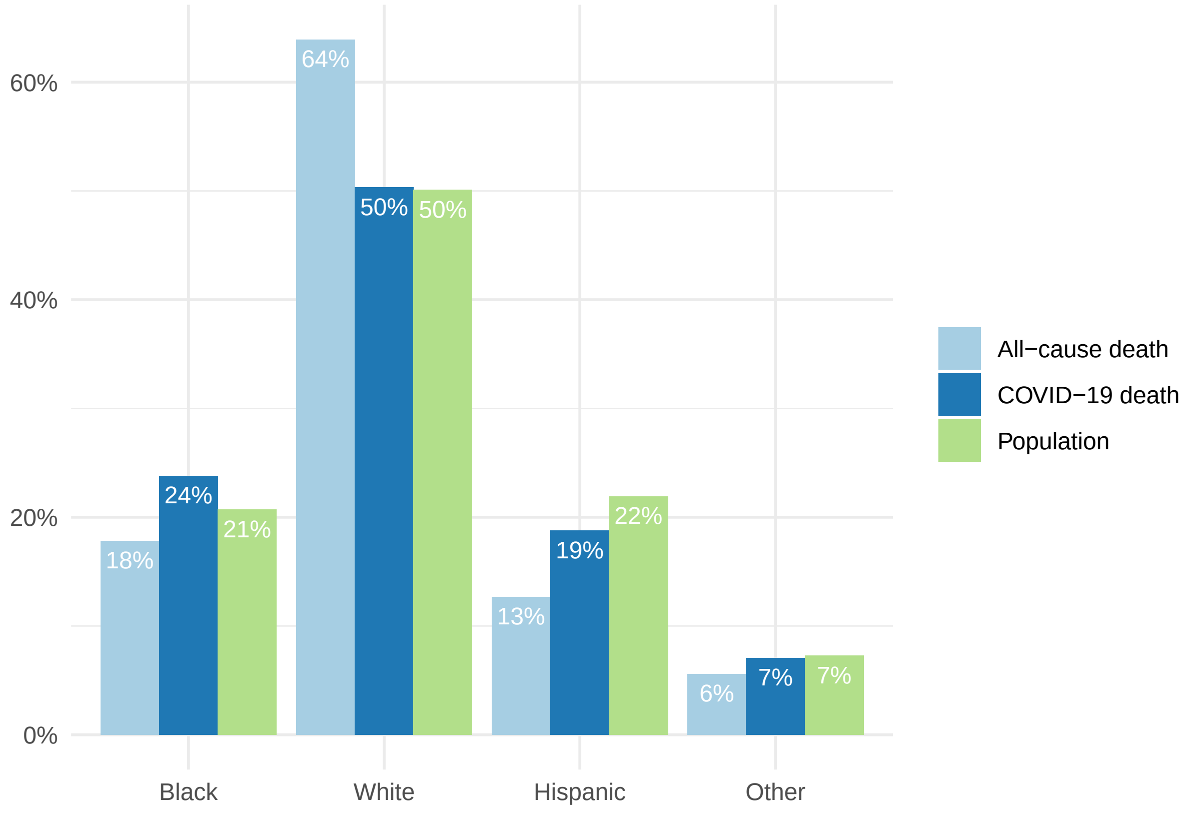


```
ggsave("gr11.png",width=7, height=3, unit="in")
```

Proportions of Non-Hispanic Blacks

Histograms (Figure 4)

The following code will reproduce Figure 4 in the manuscript that shows the percentage of Black residents by county. We use the popular `ggplot` function from the `ggplot2` package to generate the graphic and then save the results into a png file using `ggsave`.

```
df3$black_pct2 = log(df3$black_pct + 0.01)
gr1a = ggplot(df3, aes(x = black_pct)) + geom_histogram(bins = 30) +
    labs(x = "", y = "") + theme(plot.margin = unit(c(0.2,
    0.2, -0.2, -0.2), "cm"))
ggsave("gr1a.png", width = 4, height = 2, unit = "in")
gr1b = ggplot(df3, aes(x = black_pct2)) + geom_histogram(bins = 30) +
    labs(x = "", y = "") + theme(plot.margin = unit(c(0.2,
    0.2, -0.2, -0.2), "cm"))
ggsave("gr1b.png", width = 4, height = 2, unit = "in")
gr1a + gr1b
```

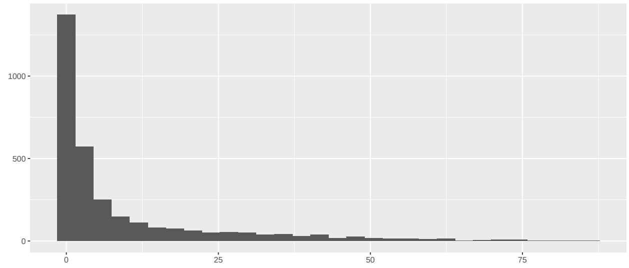

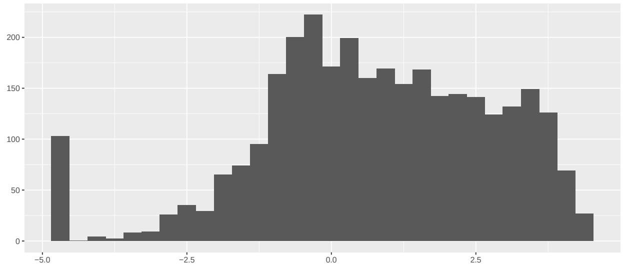

Maps (Figures 3 and 5)

The package `leaflet` is used to produce interactive graphics. The following code will reproduce Figure aa of the manuscript which shows the percentage of Black residents by US county. A popup is also used to show various information for the county selected.

Figure 3

```
states = aggregate(df3, by = list(ID = df3$state_abbr),
    FUN = unique, dissolve = T)
pal.black <- colorNumeric(palette = "plasma", domain = df3$black_pct)
mypopup = paste0("<strong>", paste(df3$name, df3$state_abbr,
    sep = ", "), "</strong>", "<br><strong>% Black: </strong>",
    round(df3$black_pct, 1), "<br><strong>Total Pop: </strong>",
    df3$total_population)
gr2 = df3 %>% leaflet(options = leafletOptions(attributionControl = FALSE)) %>%
    setView(-99, 38, zoom = 4) %>% addTiles() %>% addPolygons(weight = 0.2,
    color = "black", fillColor = ~pal.black(black_pct),
    fillOpacity = 0.5, popup = mypopup) %>% addPolylines(data = states,
    color = "black", weight = 1) %>% addLegend("bottomleft",
    title = "% Black", pal = pal.black, values = ~black_pct,
    labFormat = labelFormat(suffix = "%"))
gr2
```

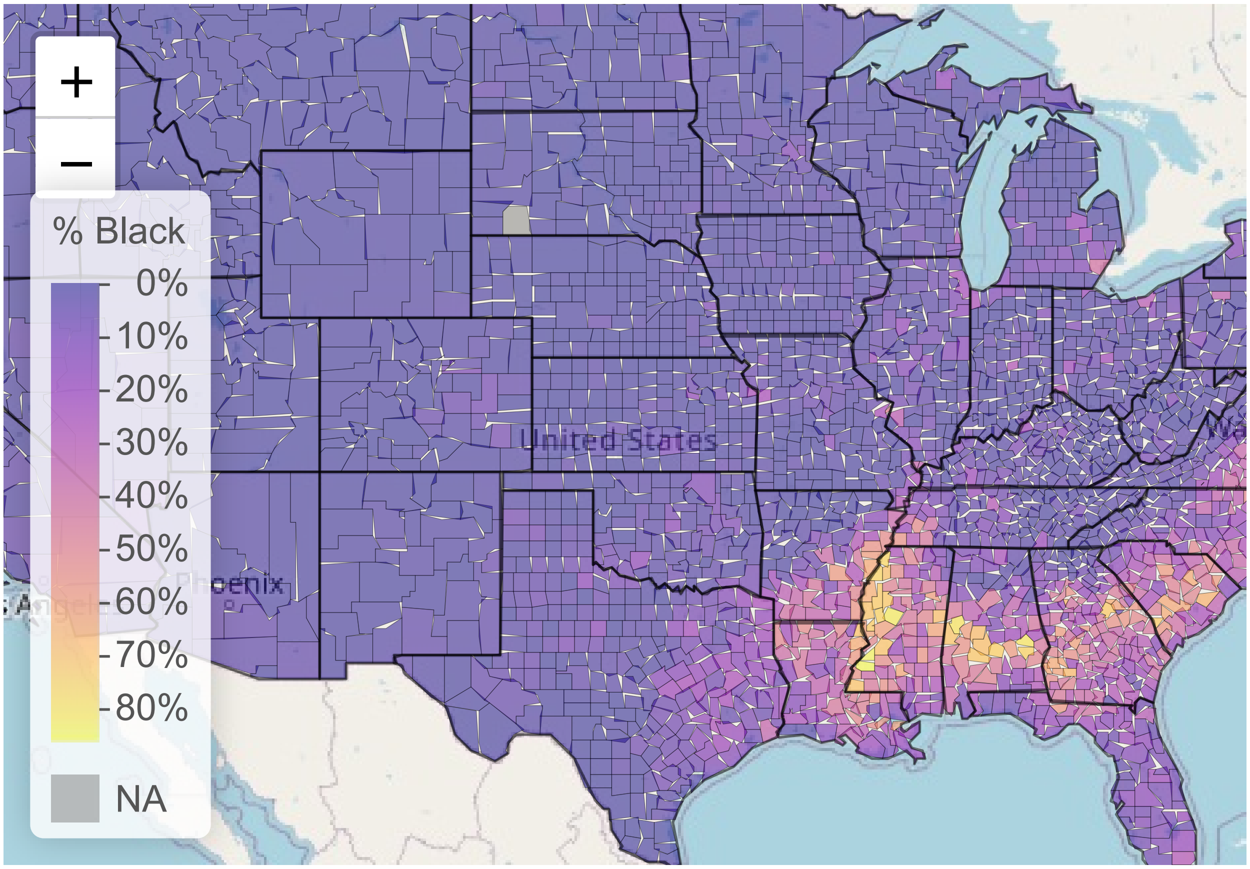


```
# mapshot(gr2,url='gr2.html')
# webshot2::webshot('gr2.html',file='gr2.png',vwidth=740,vheight=380)
```

Figure 5

The following code will reproduce Figure 5 of the manuscript which shows the percentage of Black residents by county using a non-linear color scale.

```
pal.black2 <- colorNumeric(palette = "plasma", domain = df3$black_pct2)
mypopup = paste0("<strong>", paste(df3$name, df3$state_abbr,
    sep = ", "), "</strong>", "<br><strong>% Black:  </strong>",
    round(df3$black_pct, 1), "<br><strong>Total Pop: </strong>",
    df3$total_population)
gr3 = df3 %>% leaflet(options = leafletOptions(attributionControl = FALSE)) %>%
    setView(-99, 38, zoom = 4) %>% addTiles() %>% addPolygons(weight = 0.2,
    color = "black", fillColor = ~pal.black2(black_pct2),
    fillOpacity = 0.5, popup = mypopup) %>% addPolylines(data = states,
    color = "black", weight = 1) %>% addLegend("bottomleft",
    title = "% Black", pal = pal.black2, values = ~black_pct2,
    labFormat = labelFormat(suffix = "%", transform = function(x) {
        round(exp(x) - 0.01, 1)
    }))
gr3
```

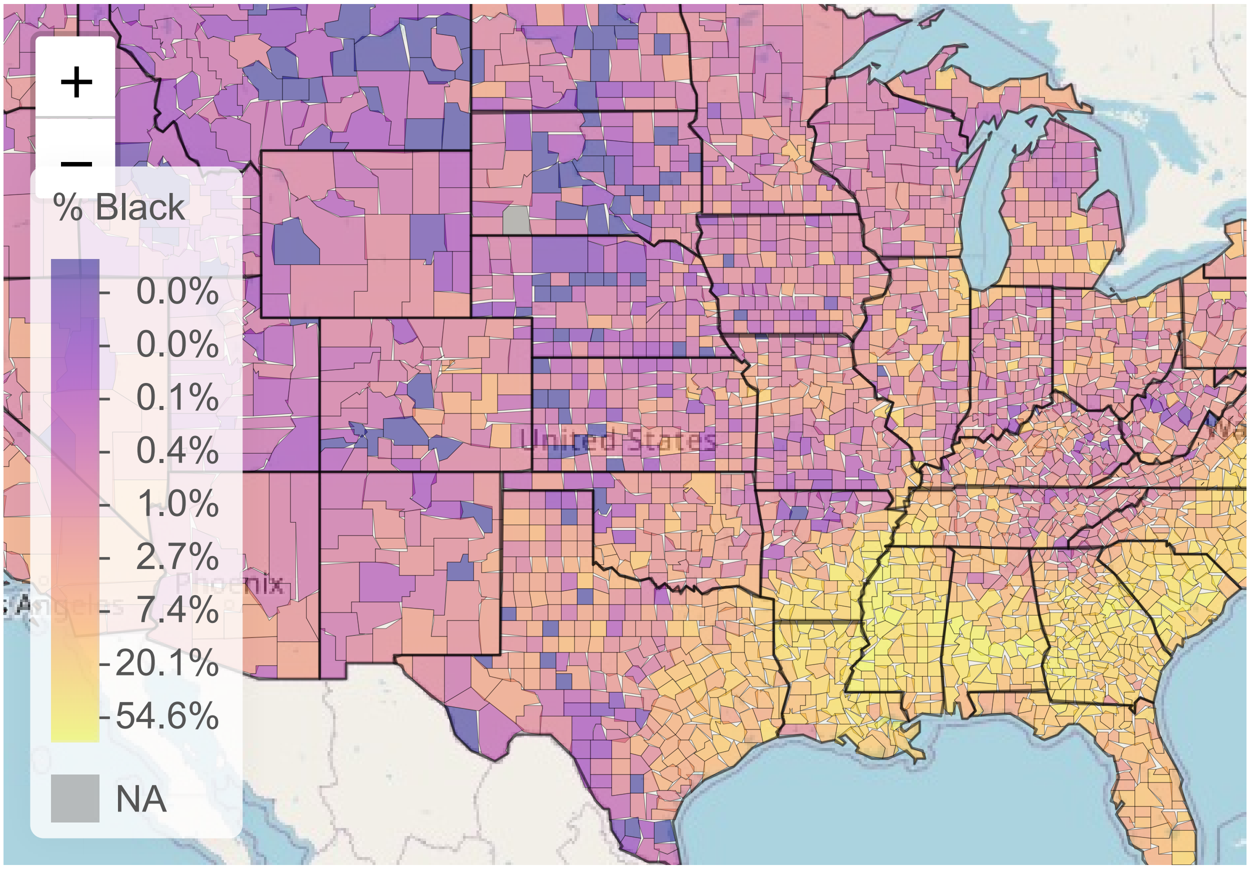


```
# mapshot(gr3,url='gr3.html')
# webshot2::webshot('gr3.html',file='gr3.png',vwidth=740,vheight=380)
```

Prevalence of COVID-19 Deaths

Histograms (Figure 6)

The following code will reproduce Figure 6 in the manuscript which shows the COVID death rates (per 10,000 residents) by US county.

```
df3[is.na(df3$deaths), "deaths"] = 0
df3$death_rate = df3$deaths/df3$total_population * 10000
df3$death_rate2 = log(df3$deaths/df3$total_population *
    10000 + 0.1)
gr4a = ggplot(df3, aes(x = death_rate)) + geom_histogram(bins = 30) +
    labs(x = "", y = "") + theme(plot.margin = unit(c(0.2,
    0.2, -0.2, -0.2), "cm"))
ggsave("gr4a.png", width = 4, height = 2, unit = "in")
gr4b = ggplot(df3, aes(x = death_rate2)) + geom_histogram(bins = 30) +
    labs(x = "", y = "") + theme(plot.margin = unit(c(0.2,
    0.2, -0.2, -0.2), "cm"))
ggsave("gr4b.png", width = 4, height = 2, unit = "in")
gr4a + gr4b
```

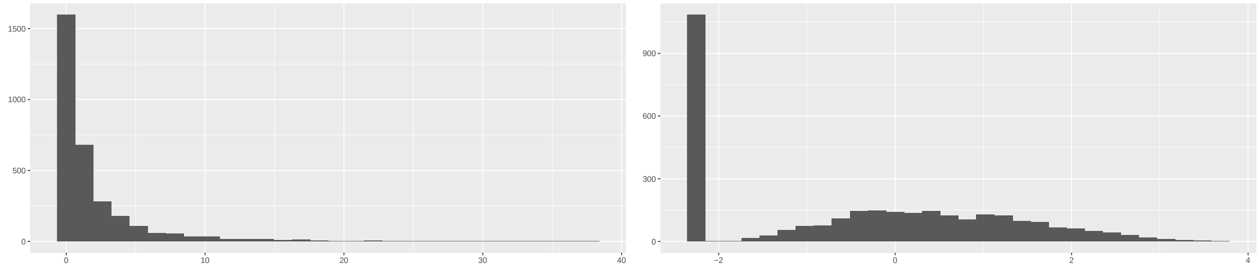

Maps (Figure 7)

Figure 7a

The following code will reproduce Figure 7a in the manuscript which shows COVID death rates (per 10,000 residents) by county.

```
pal.covid.deaths <- colorNumeric(palette = "Reds", domain = df3$death_rate)
pal.covid.deaths2 <- colorNumeric(palette = "Reds", domain = df3$death_rate2)
mypopup = paste0("<strong>", paste(df3$name, df3$state_abbr,
    sep = ", "), "</strong>", "<br><strong>Death rate (per 10k):  </strong>",
    round(df3$death_rate, 1), "<br><strong>Total Pop: </strong>",
    df3$total_population)
gr5 = df3 %>% leaflet(options = leafletOptions(attributionControl = FALSE)) %>%
    setView(-99, 38, zoom = 4) %>% addTiles() %>% addPolygons(weight = 0.2,
    color = "black", fillColor = ~pal.covid.deaths(death_rate),
    fillOpacity = 0.5, popup = mypopup) %>% addPolylines(data = states,
    color = "black", weight = 1) %>% addLegend("bottomleft",
    title = "COVID Deaths<br>per 10,000", pal = pal.covid.deaths,
    values = ~death_rate)
css_fix <- "div.info.legend.leaflet-control br {clear: both;}"  # CSS to correct spacing
html_fix <- htmltools::tags$style(type = "text/css", css_fix)  # Convert CSS to HTML
gr5 %>% htmlwidgets::prependContent(html_fix)
```

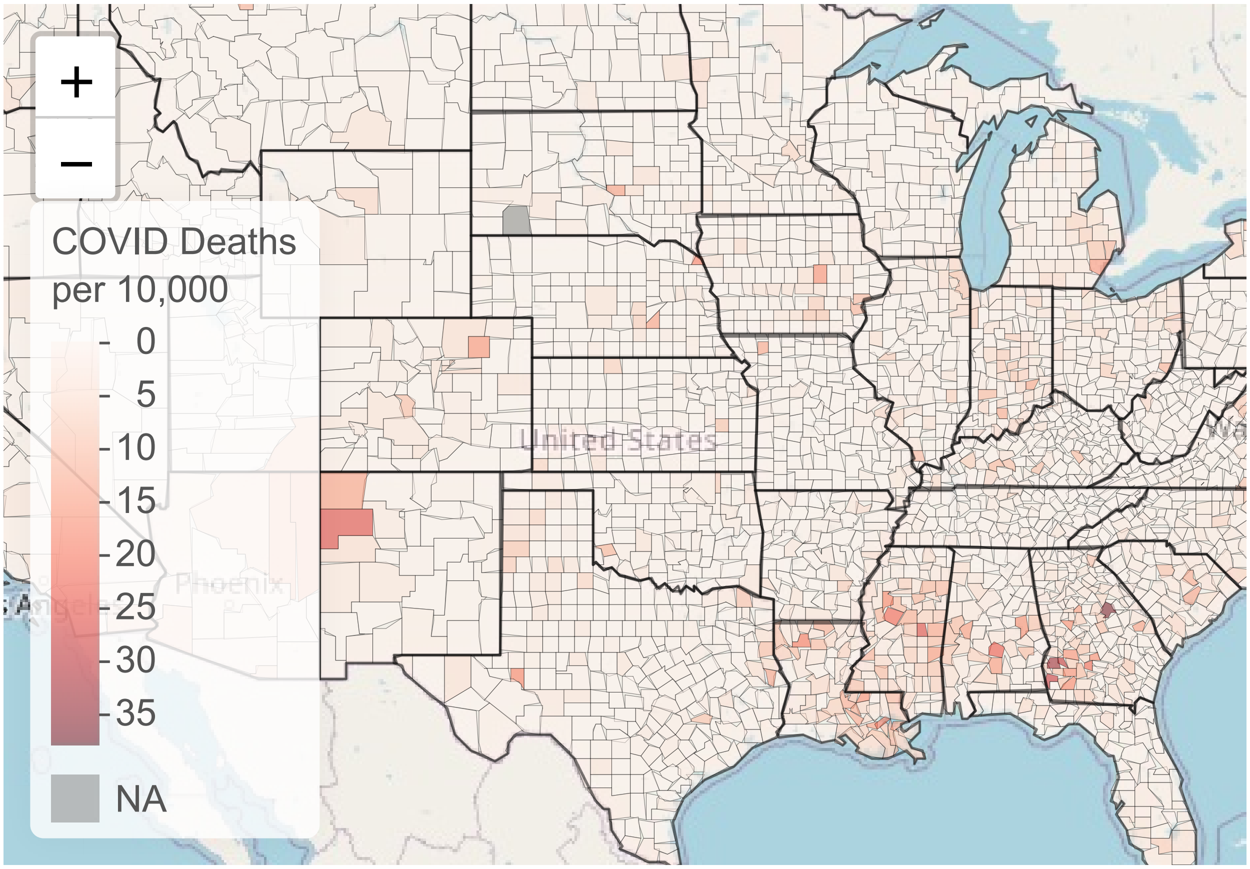


```
# mapshot(gr5%>% htmlwidgets::prependContent(html_fix)
# ,url='gr5.html')
# webshot2::webshot('gr5.html',file='gr5.png',vwidth=740,vheight=380)
```

Figure 7b

The following code will reproduce Figure 7b in the manuscript which shows COVID death rates (per 10,000 residents) by county using a non-linear color scale.

```
mypopup = paste0("<strong>", paste(df3$name, df3$state_abbr,
    sep = ", "), "</strong>", "<br><strong>Death rate (per 10k): </strong>",
    round(df3$death_rate, 1), "<br><strong>Total Pop: </strong>",
    df3$total_population)
gr6 = df3 %>% leaflet(options = leafletOptions(attributionControl = FALSE)) %>%
    setView(-99, 38, zoom = 4) %>% addTiles() %>% addPolygons(weight = 0.2,
    color = "black", fillColor = ~pal.covid.deaths2(death_rate2),
    fillOpacity = 0.5, popup = mypopup) %>% addPolylines(data = states,
    color = "black", weight = 1) %>% addLegend("bottomleft",
    title = "COVID Deaths<br>per 10,000", pal = pal.covid.deaths2,
    values = ~death_rate2, labFormat = labelFormat(transform = function(x) {
        round(exp(x) - 0.1, 1)
    }))
gr6 %>% htmlwidgets::prependContent(html_fix)
```

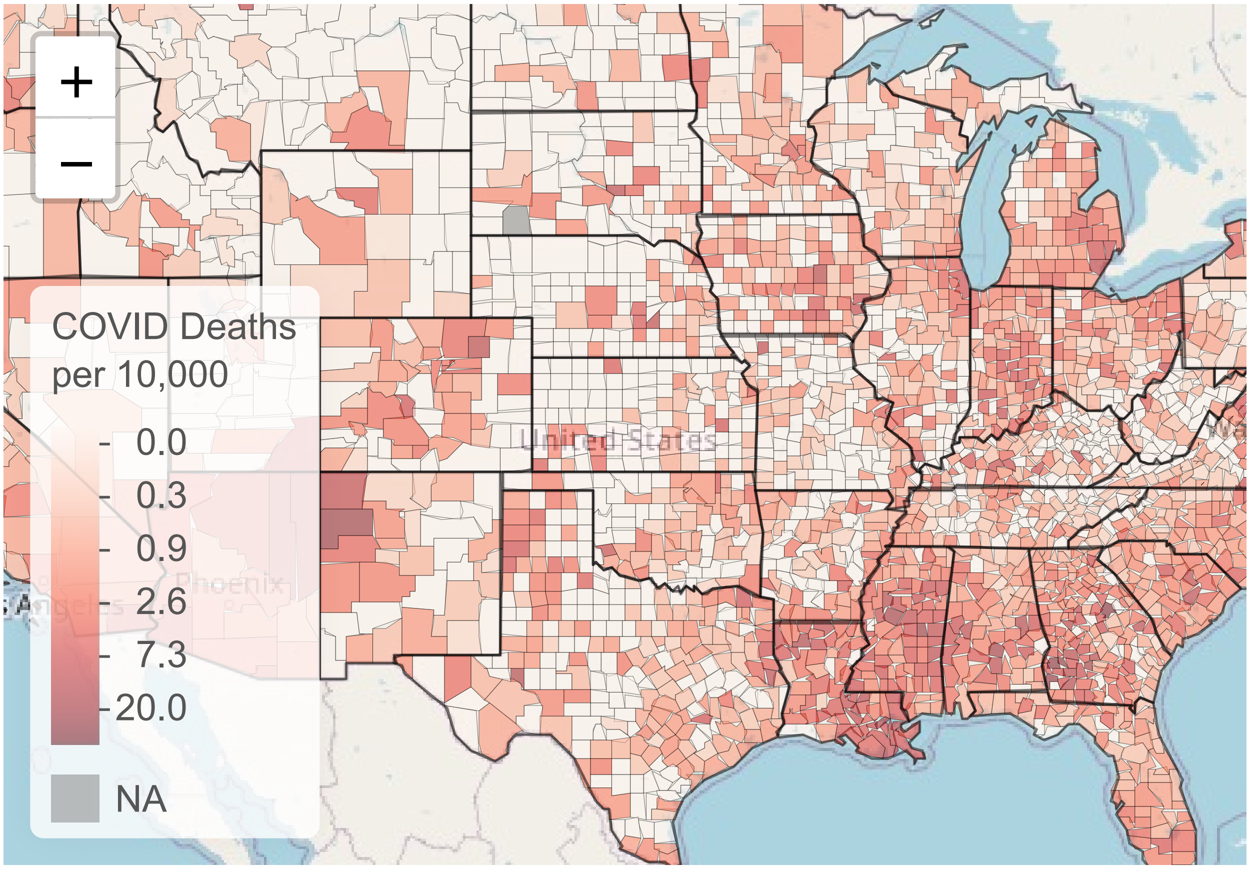


```
# mapshot(gr6%>% htmlwidgets::prependContent(html_fix)
# ,url='gr6.html')
# webshot2::webshot('gr6.html',file='gr6.png',vwidth=740,vheight=380)
```

Population Density

Adding land area variable

- Land areas for each county is contained in the file `LND01.xls` and can be downloaded from the US Census Bureau. Having the land areas for each county allows us to calculate the average population density for each county. The dataset can be accessed from this US Census website: https://www.census.gov/library/publications/2011/compendia/usa-counties-2011.html#LND. We select the two columns of interest from the land dataset (fips code and land area) and then merge with our previous datasets into `df4`.

```
data.land <- read.csv("Data/Land.csv")
data.land <- data.land[, c(2, 24)]
data.land <- dplyr::rename(data.land, c(fips = "STCOU",
    area = "LND110210D"))
data.land$fips <- as.numeric(data.land$fips)
df4 = merge(df3, data.land, by = "fips", all.x = TRUE)
```

Histograms

The following code will produce histograms that show the spatial distribution of population densities by county

```
df4$density = df4$total_population/df4$area
df4$density2 = log(df4$total_population/df4$area + 0.01)
gr7a = ggplot(df4, aes(x = density)) + geom_histogram(bins = 30) +
    labs(x = "", y = "") + theme(plot.margin = unit(c(0.2,
    0.2, -0.2, -0.2), "cm"))
ggsave("gr7a.png", width = 4, height = 2, unit = "in")
gr7b = ggplot(df4, aes(x = density2)) + geom_histogram(bins = 30) +
    labs(x = "", y = "") + theme(plot.margin = unit(c(0.2,
    0.2, -0.2, -0.2), "cm"))
ggsave("gr7b.png", width = 4, height = 2, unit = "in")
gr7a + gr7b
```

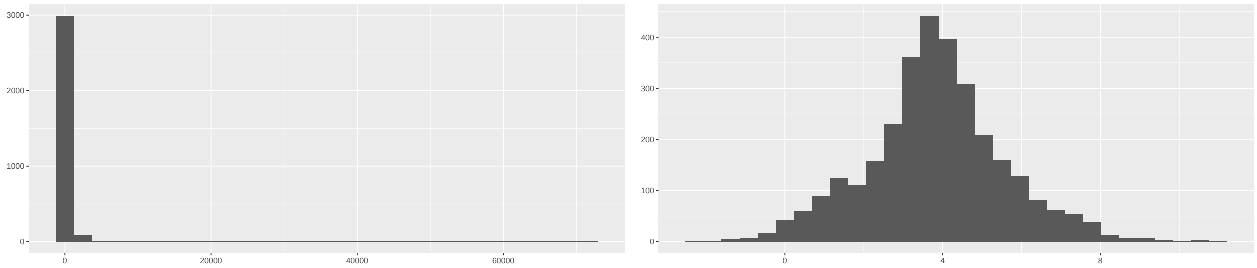

Maps (Figure 8)

The following code will produce an interactive map that shows the spatial distribution of population densities by county.

```
pal.density <- colorNumeric(palette = "Greens", domain = df4$density)
pal.density2 <- colorNumeric(palette = "Greens", domain = df4$density2)
mypopup = paste0("<strong>", paste(df3$name, df3$state_abbr,
    sep = ", "), "</strong>", "<br><strong>Population Density: </strong>",
    round(df4$density, 1), "<br><strong>Total Pop: </strong>",
    df3$total_population)
gr8 = df4 %>% leaflet(options = leafletOptions(attributionControl = FALSE)) %>%
    setView(-99, 38, zoom = 4) %>% addTiles() %>% addPolygons(weight = 0.2,
    color = "black", fillColor = ~pal.density(density),
    fillOpacity = 0.5, popup = mypopup) %>% addPolylines(data = states,
    color = "black", weight = 1) %>% addLegend("bottomleft",
    title = "Population<br>per sq mile", pal = pal.density,
    values = ~density)
gr8 %>% htmlwidgets::prependContent(html_fix)
```

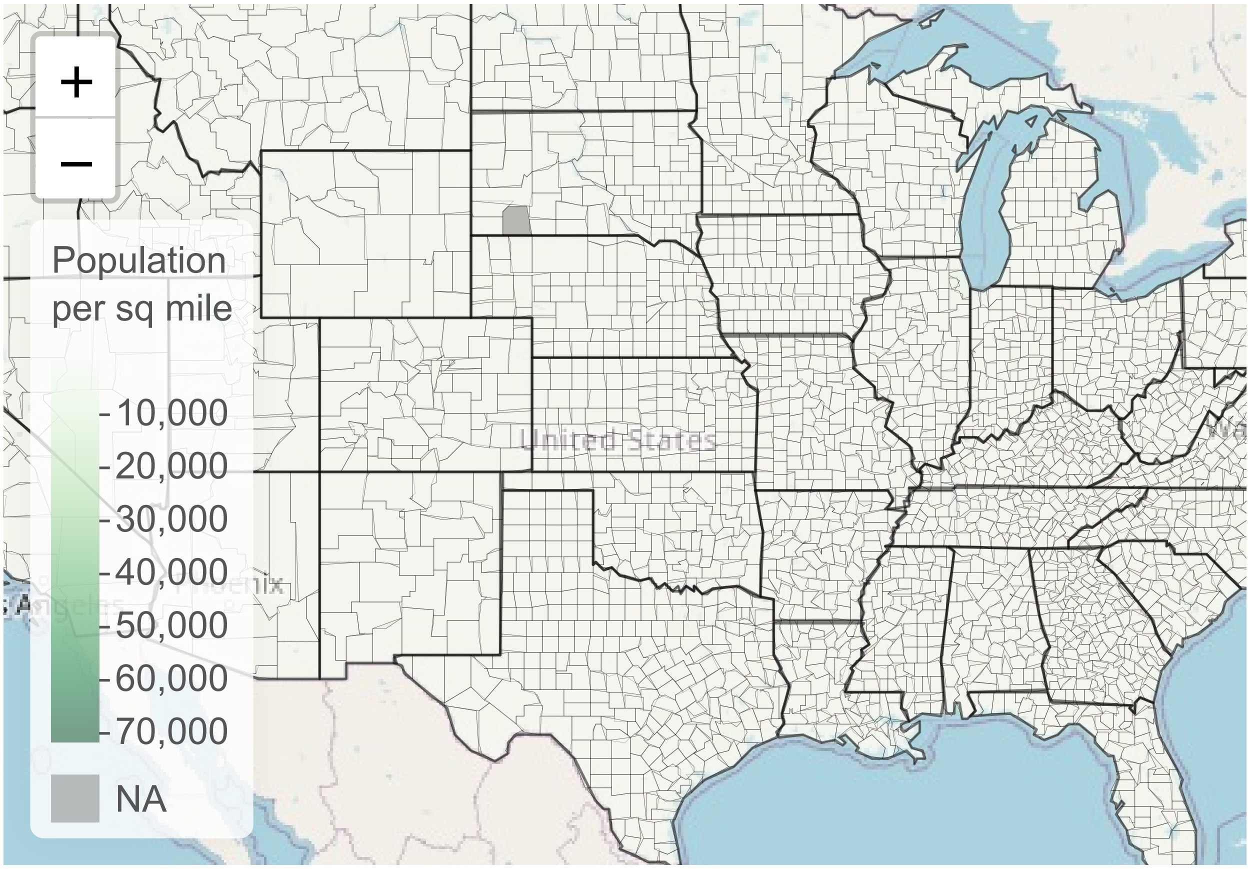


```
# mapshot(gr8%>% htmlwidgets::prependContent(html_fix)
# ,url='gr8.html')
# webshot2::webshot('gr8.html',file='gr8.png',vwidth=740,vheight=380)
```

Figure 8

The following code will reproduce Figure 8 in the manuscript which shows the spatial distribution of population densities by county using a non-linear color scale.

```
mypopup = paste0("<strong>", paste(df3$name, df3$state_abbr,
    sep = ", "), "</strong>", "<br><strong>Population Density: </strong>",
    round(df4$density, 1), "<br><strong>Total Pop: </strong>",
    df3$total_population)
gr9 = df4 %>% leaflet(options = leafletOptions(attributionControl = FALSE)) %>%
    setView(-99, 38, zoom = 4) %>% addTiles() %>% addPolygons(weight = 0.2,
    color = "black", fillColor = ~pal.density2(density2),
    fillOpacity = 0.5, popup = mypopup) %>% addPolylines(data = states,
    color = "black", weight = 1) %>% addLegend("bottomleft",
    title = "Population<br>per sq mile", pal = pal.density2,
    values = ~density2, labFormat = labelFormat(transform = function(x) {
        round(exp(x) - 0.01, 0)
    }))
gr9 %>% htmlwidgets::prependContent(html_fix)
```

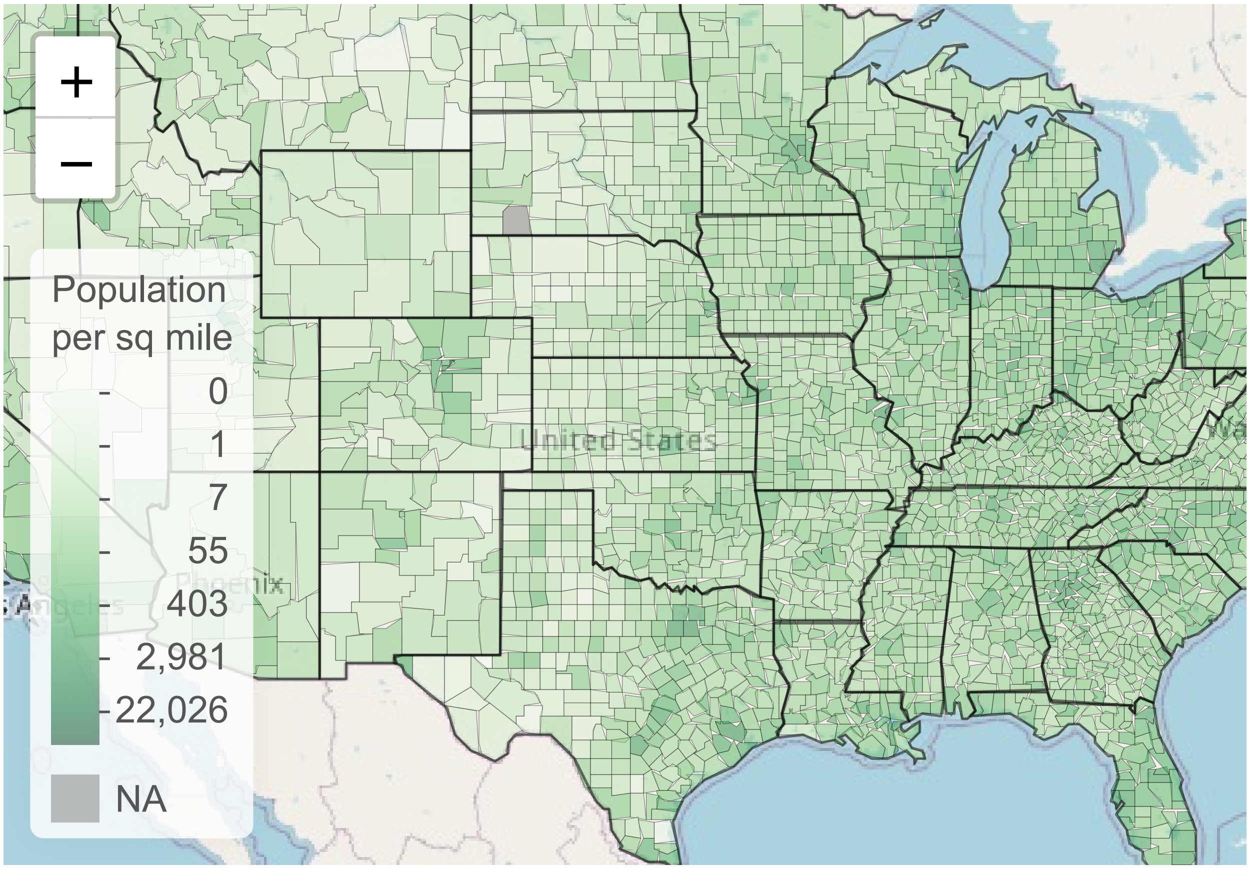


```
# mapshot(gr9%>% htmlwidgets::prependContent(html_fix)
# ,url='gr9.html')
# webshot2::webshot('gr9.html',file='gr9.png',vwidth=740,vheight=380)
```

Boxplot

The following code will reproduce Figure 8 in the manuscript. We first divide the counties into 4 groups by their population density. The first boxplot shows the percentage of Black residents across different population densities and the second boxplot shows the COVID death rates across population densities. The purpose of these diagrams is to detect any potential confounding due to population densities.

```
df4$density.binned = cut(df4$density, quantile(df4$density,
    prob = 0:4/4, names = FALSE, na.rm = TRUE))
df5 = df4 %>% select(density.binned, black_pct2, death_rate2) %>%
    as_tibble() %>% tidyr::drop_na()
gr9a = ggplot(df5, aes(x = density.binned, y = black_pct2)) +
    geom_boxplot() + labs(x = "Population Density", y = "Log-transformed % Black") +
    theme(plot.margin = unit(c(0.2, 0.2, 0.2, 0.2), "cm")) +
    scale_x_discrete(labels = c("Very Low", "Low", "High",
        "Very High"))
gr9a
```

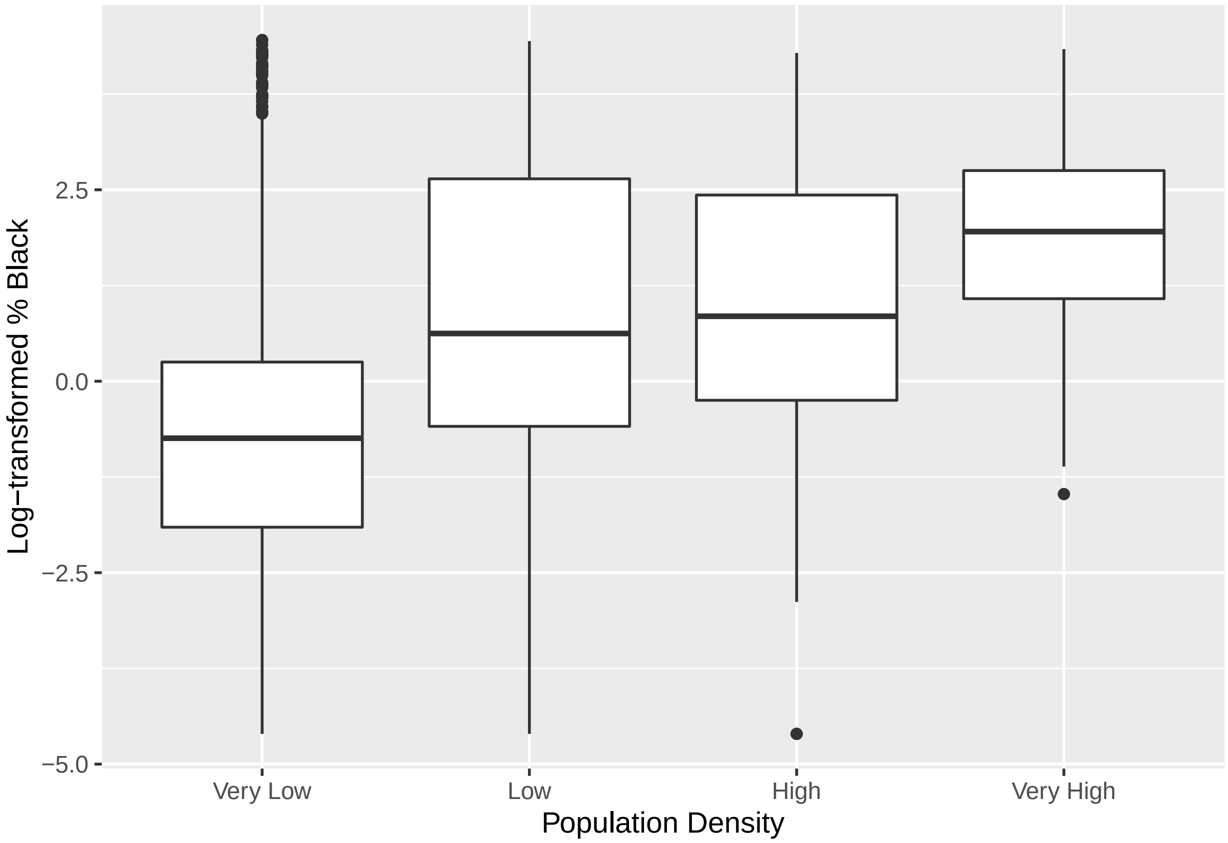


```r
ggsave("gr9a.png", width = 4, height = 3, unit = "in")
gr9b = ggplot(df5, aes(x = density.binned, y = death_rate2)) +
    geom_boxplot() + labs(x = "Population Density", y = "Log-transformed Covid Death Rate") +
    theme(plot.margin = unit(c(0.2, 0.2, 0.2, 0.2), "cm")) +
    scale_x_discrete(labels = c("Very Low", "Low", "High",
        "Very High"))
gr9b
```

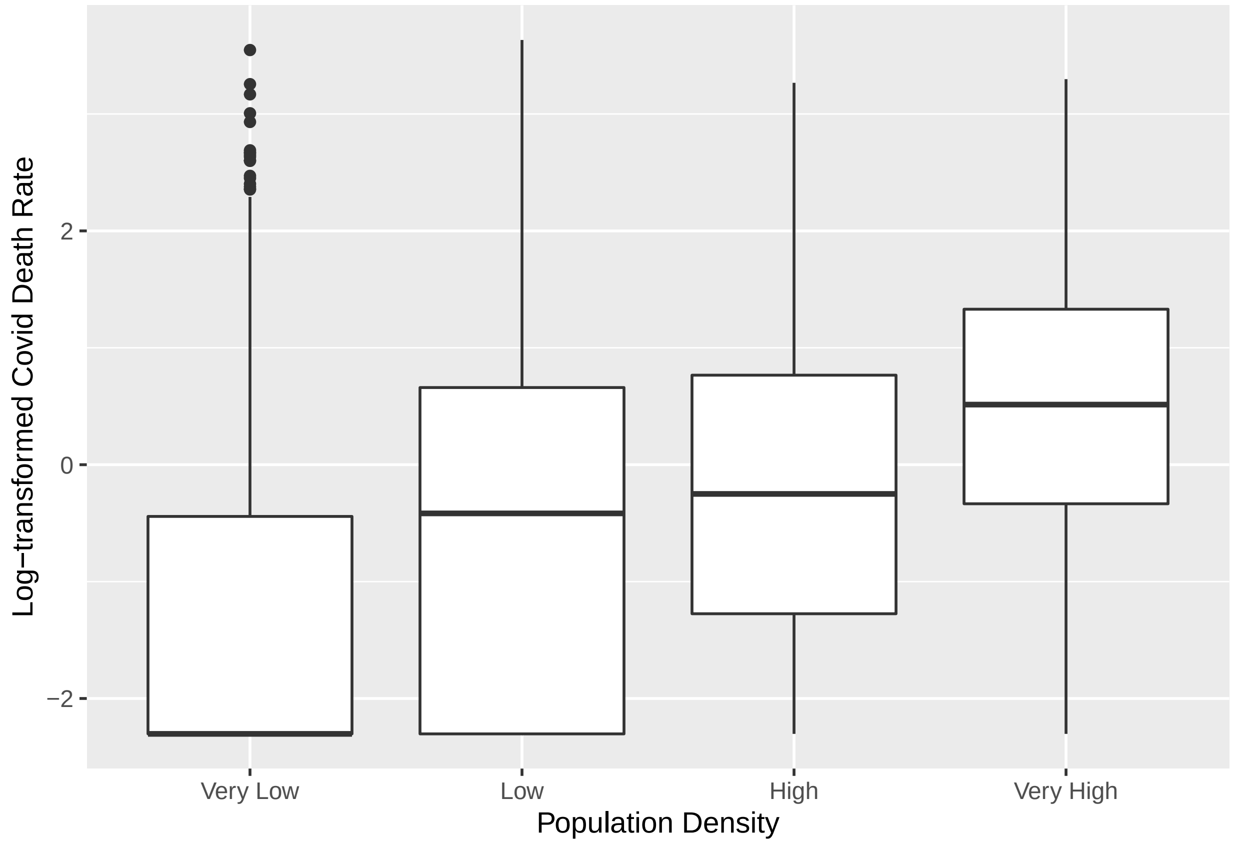


```
ggsave("gr9b.png", width = 4, height = 3, unit = "in")
```
